# Antidepressant activity of flavones from traditional Chinese medicine: a meta-analysis

**DOI:** 10.1080/13880209.2025.2467374

**Published:** 2025-02-25

**Authors:** Qing Wang, Youyuan Lu, Xue Mi, Caiyan Yang, Wei Ma, Changbo Xia, Hanqing Wang

**Affiliations:** a College of Pharmacy, Ningxia Medical University, Yinchuan, China; b Department of Pharmacy, The First People’s Hospital of Yinchuan, Yinchuan, China; c Ningxia Regional Characteristic Traditional Chinese Medicine Collaborative Innovation Center Co-constructed by the Province and Ministry, Ningxia Engineering and Technology Research Center for Modernization of Regional Characteristic Traditional Chinese Medicine, Ningxia Medical University, Yinchuan, China; d Department of Pharmacy, Central’s Hospital of Xinxiang, Xinxiang, China; e Key Laboratory of Ningxia Minority Medicine Modernization, Ministry of Education, Ningxia Medical University, Yinchuan, China

**Keywords:** Depression, flavones, animal studies, meta-analysis, mechanisms

## Abstract

**Context:**

Flavones, the key active components in Traditional Chinese Medicine (TCM), have demonstrated antidepressant activity. Given the numerous animal studies conducted, a systematic analysis is essential to provide a valuable reference for future research.

**Object:**

This study investigated the antidepressant activity of flavones based on animal models and summarized the underlying mechanisms.

**Methods:**

We systematically searched 7 bibliographic Databases as of August 12, 2023, such as Web of Science, PubMed, China National Knowledge Infrastructure, etc. The meta-analysis was performed using either the random or fixed-effect model, supplemented by trial sequential analysis (TSA). The Grading of Recommendations, Assessment, Development and Evaluations (GRADE) approach was used to assess the quality of evidence.

**Results:**

A total of 25 studies involving 458 mice were included, identifying five flavones (baicalin, baicalein, apigenin, luteolin, vitexin) with antidepressant activity. Compared to the control group, flavones significantly reduced immobility time in forced swimming and tail suspension tests. Flavones also decreased serum and hippocampal levels of interleukin (IL)-1β and tumor necrosis factor-alpha (TNF-α), reduced nuclear factor kappa B (NF-κB) levels, and increased brain-derived neurotrophic factor (BDNF) levels. Relative to the positive group, flavones raised IL-6, sucrose preference rate, and corticosterone (CORT) levels, with no significant differences in other factors. The TSA showed the efficacy of flavones for treating depression with adequate ‘information size’ for the primary outcome.

**Conclusions:**

The results demonstrate that flavones exert protective effects against depression in mice, primarily by stimulating neurotrophic factors and modulating inflammatory pathways. These findings emphasize their potential as promising candidates for the development of novel antidepressant therapies.

## Introduction

Traditional Chinese Medicine (TCM) is one of the most ancient healing systems, boasting a rich history of over two millennia of accumulated knowledge and practical application. TCM is considered a complementary or alternative medical system in most Western countries. It is estimated that millions of patients worldwide use TCM or related practices (Li 2022), ranging from treating the common cold to chronic diseases such as diabetes and ischemic stroke (Shen and Yin 2021; Zhu et al. 2022). It is also worth emphasizing that many contemporary pharmaceuticals originate from herbal and natural sources. For instance, substances like St. John’s wort, curcumin, and oligosaccharides in Morindae Officinalis Radix have demonstrated efficacy in addressing mild depression.

Depression is the most common life-threatening and debilitating mental disorder that features abnormalities in mode and manifests as depressed mood, fatigue, irritability, cognitive impairment, and loss of interest and joy (Hammen 2018). According to the World Health Organization, 300 million people worldwide suffer from depression. Notably, depression has a deleterious impact on human capital, interpersonal interactions, and social activities. It also poses an elevated risk of mortality due to suicide and other comorbid conditions (Herrman et al. 2019). In contemporary medical practice, conventional antidepressant medications are widely used to treat depression. These medications encompass a range of classes, including tricyclic antidepressants (TCA), monoamine oxidase inhibitors (MAOIs), selective ­serotonin reuptake Inhibitors (SSRIs), and selective 5-hydroxytryptamine (5-HT) reuptake inhibitors. Nevertheless, certain antidepressant drugs exhibit limited effectiveness in treating individuals who are resistant to standard therapies and are associated with significant adverse effects. These adverse effects encompass gastrointestinal disturbances, cardiovascular complications, headaches, and fatigue, which significantly impact patients’ quality of life and adherence to medication regimens (Solmi et al. 2020). Therefore, new treatments must be urgently needed to develop novel therapeutic agents with more effective and fewer side effects to treat depression.

Flavones, which possess a C6-C3-C6 skeleton, mainly originate from plant metabolism and have demonstrated beneficial effects on various aspects of human physiology and health, such as antitumor, antiplatelet, anti-malarial, anti-inflammatory, anticonvulsant, and antidepressant properties (Khan et al. 2018; Ko et al. 2020; Melrose 2023). TCM has made substantial strides in the treatment of depression while minimizing adverse effects. Numerous animal experiments have been conducted to investigate the antidepressant properties of flavones in the context of depression. Previous meta-analyses (Jia et al. 2023), encompassing a limited number of participants and studies, have suggested that flavonoids may alleviate symptoms of depression and anxiety, specifically targeting clinical patient populations. Additionally, another study has explored the antidepressant mechanisms attributed to flavonoids (Lysrayane et al. 2021). Consequently, this study will undertake a systematic assessment of the effectiveness of flavones in depression-related animal models, thereby offering prospective avenues for subsequent clinical trials.

## Materials and methods

In this study, we complied with the Preferred Reporting Items for Systematic Reviews and Meta-Analyses (PRISMA) reporting statement (Page et al. 2021) and registered the study on the International Prospective Register of Systematic Reviews (registration no. CRD42023416795). The PRISMA checklists are available in Appendix Files, Table S1.

### Search strategy

A total of seven databases were searched from their inception until March 16, 2023.The English databases include PubMed, Embase, Web of Science and the Chinese databases include Sinomed, China National Knowledge Infrastructure (CNKI), China Science and Technology Journal Database, and Wanfang. A combination of MeSH terms, Emtree headings, and keywords was used for ‘depression’, ‘mice’, ‘apigenin’, ‘luteolin’, ‘baicalin’, ‘vitexin’, and ‘baicalein’. The search strategy is presented in Appendix Files, Table S2.

### Study selection

Two investigators (W.Q. and M.X.) independently assessed the titles and retrieved the full-text studies in accordance with the inclusion criteria. The inclusion principles are as follows: (i) Animals: Mouse models of depression, without restrictions on age, sex, or duration of disease induction; (ii) Interventions: Treatment with flavone components, without limitations on administration methods, frequency, and dosage; (iii) Two comparison groups were used in this study. One is the model comparison, which was treated with a vehicle, distilled water, or saline after successful depression modeling. The other is the positive medicine comparison, treated with antidepressant drugs after successful depression modeling; (iv) Outcomes: The primary outcomes were behavioral tests of animals, including the tail suspension test (TST), forced swimming test (FST), open field test (OFT), and the sucrose preference test (SPT). The secondary outcomes included pro-inflammatory cytokines, such as interleukin-1β (IL-1β) levels, interleukin-6 (IL-6) levels, tumor necrosis factor-alpha (TNF-α) levels, serum corticosterone levels, brain-derived neurotrophic factor (BDNF) contents, and the level of nuclear factor kappa B (NF-κB); (v) Studies: The study design included controlled *in vivo* experiments with a control group, but was not restricted to randomized controls. The exclusion criteria were as follows: (i) studies lacking data from the depression model group or the antidepressant drugs group; (ii) studies involving flavone combinations with other compounds and treatments or association with other interventions; (iii) studies with duplicate publication or that failed to provide the required outcomes; (iv) studies that were conducted *in vitro*.

### Data extraction

The data, including the characteristics of studies, model, sex, weight, age, number of animals included, dose, time of intervention, and method of administration, was extracted from the included studies by two investigators independently. We first attempted to extract data from tables or figures; when these were not reported, we attempted to extract data from the bar plots of each study through the WebPlotDigitizer program. To comprehensively elucidate the efficacy of all flavones, we selected a regimen involving a higher dosage. We resolved discrepancies through discussion and, if necessary, adjudication by a third investigator (L.Y.Y).

### Quality assessment

The risk of bias was independently evaluated by W.Q and M.X using the Systematic Review Center for Laboratory Experimentation (SYRCLE) Risk of Bias tool (Hooijmans et al. 2014). If discrepancies existed, L.Y.Y participated in the evaluation. The domains assessed included sequence generation, baseline characteristics, allocation concealment, random housing, blinding (performance bias), random outcome assessment, blinding (detection bias), incomplete outcome data, selective outcome reporting, and other sources of bias. Assessment results were categorized as ‘low risk of bias’, ‘high risk of bias’, and ‘unclear risk of bias’.

### Statistical analysis

We performed the meta-analysis using RevMan 5.4.1 and Stata 14.0. Mean differences (MDs) with 95% confidence intervals (95% CIs) were shown for continuous data. Heterogeneity was assessed using the χ^2^ test and *I^2^* statistics. Due to the lack of significant heterogeneity (*p* ≥ 0.10, *I^2^* ≤ 50%), a fixed-effect model was used. Trial sequential analysis (TSA) was conducted with the primary outcome indicators TST, FST, SPT and OFT utilizing TSA software version 0.9.5.10 to evaluate the reliability of the evidence and to determine if the sample size has reached the desired sample size.

### Subgroup and sensitivity analysis

We conducted subgroup analyses based on the type of flavone, dose and method of administration. We assessed publication bias for the primary outcome using funnel plot asymmetry and Egger’s test, provided that more than ten studies were available. Sensitivity analyses were conducted to verify the robustness of the results by excluding one study per time and repeating the meta-analysis. Stata version 14.0 software was utilized for the publication bias and sensitivity analyses.

### GRADE evaluation

Two investigators assessed the quality of evidence using GRADE (Grading of Recommendations, Assessment, Development, and Evaluation) (Guyatt et al. 2011). We downgraded each outcome based on bias, inconsistency, indirectness, imprecision, and publication bias.

## Results

### Study selection

**Figure 1.**
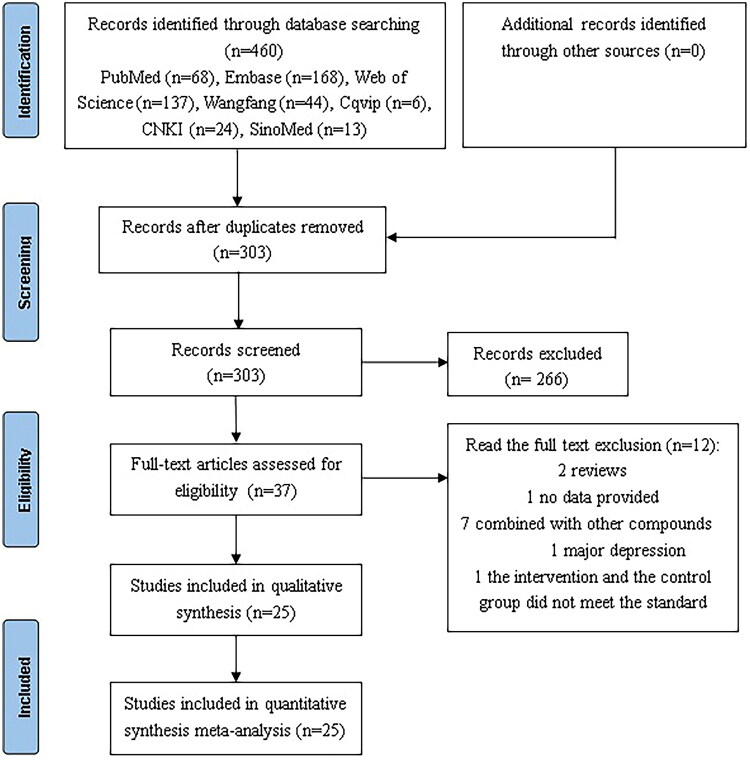
Preferred reporting items for systematic reviews and meta-analyses (PRISMA) flowchart.

After removing duplicate publications, 303 studies were screened by reviewing their titles and abstracts. Ultimately, 37 full texts were assessed for eligibility, of which 25 were selected for further study. A visual description of the study selection process in the PRISMA flowchart in Figure 1.

### Characteristics of included studies

Compared to female mice, male mice were more commonly used in these studies, with the number of animals per group ranging from 6 to 12. In these 25 literatures, a total of five depression models were investigated: chronic unpredictable mild stress (CUMS), chronic mild stress (CMS), corticosterone (CORT), chronic restraint stress (CRS), and lipopolysaccharide-induced depression. Among these, the CUMS model was the most frequently used. Additionally, two of the included studies did not explicitly describe the depression model used (Can et al. 2013; Al-Yamani et al. 2022). For the flavone components with antidepressant activity, baicalin, baicalein, apigenin, luteolin, and vitexin were reported, with baicalin receiving the most attention. Typically, mice were orally administered flavones and antidepressant drugs. The characteristics of the included studies are summarized in Table 1.

**Table 1.** Characteristics of the included studies.

References	Modeling	Sex and Species/	Group (n)	Weight (g)/age (w)	Intervention Measures			Drug delivery method	Outcomes
					Model	Positive	Flavones		
Can et al. 2013	NR	Male BALB/c	8	30–35/6	NR	Reb (20 mg/kg) once	Vit (30 mg/kg) once	i. g.	*a*
Liu et al. 2013	CUMS	Male ICR	10	18–20/NR	NR + CUMS 6w	NR	Lut (6 mg/kg) for 21d + CUMS 6w	i. g.	*a, b*
Li et al. 2015a	LPS	Male ICR	10	18–22/NR	Sal for 7d + LPS (0.5 mg/kg/d) for 7d	Flu (20 mg/kg/d) for 7d + LPS (0.5 mg/kg/d) for 7d	Api (50 mg/kg/d) for 7d + LPS (0.5 mg/kg/d) for 7d	i. p.	*a, c, d, e, f, i*
Li et al. 2015b	CORT	Male ICR	10	18–22/NR	CORT (40 mg/kg/d) for 3w	Flu (20 mg/kg/d) for 21d + CORT (40 mg/kg/d) for 3w	BA (20 mg/kg/d) for 21d + CORT (40 mg/kg/d) for 3w	i. g.	*b, c, g, j*
Sheng et al. 2016	CORT	Male ICR	10	18–22/NR	Sal for 21d + CORT 3w	Flu (20 mg/kg/d) for 21d + CORT 3w	BA (20 mg/kg/d) for 21d + CORT 3w	i. g.	*b, c*
Weng et al. 2016	CORT	Male ICR	8	24 ± 2/NR	CORT (40 mg/kg/d) for 3w	Flu (20 mg/kg/d) for 21d + CORT (40 mg/kg/d) for 3w	Api (40 mg/kg/d) for 21d + CORT (40 mg/kg/d) 3w	i. g.	*b, c, g, j*
Guo et al. 2019	CUMS	Male ICR	12	18–22/NR	Sal for 21d + CUMS 8w	Flu (20 mg/kg/d) for 21d + CUMS 8w	BA (60 mg/kg/d) for 21d + CUMS 8w	i. g.	*a, b, c, d, e, f, h*
Liu et al. 2019a	CUMS	Male SPF ICR	12	18–22/NR	Sal for 21d + CUMS 6w	Flu (20 mg/kg/d) for 21d + CUMS 6w	BA (50 mg/kg/d) for 21d + CUMS 6w	i. g.	*a, b, c, d, e, f, h, i*
Liu et al. 2019b	CUMS	Male ICR	10	30 ± 3/NR	Sal for 21d + CUMS 3w	Flu (5.2 mg/kg/d) for 21d + CUMS 3w	BA (12 mg/kg/d) for 21d + CUMS 3w	i. g.	*b, c*
Lu et al. 2019	CMS	Male ICR	10	20–25/8	NR + CUMS 3w	Flu (5.2 mg/kg/d) for 21d + CUMS 3w	BA (100 mg/kg/d) for 21d + CUMS 3w	i. p.	*b, c*
Zhang et al. 2019a	CORT	Male C57BL/6	8	18–22/8	Sal for 28d + CUMS 8w	Flu (18 mg/kg) for 28d + CORT 8w	BA (160 mg/kg/d)for 28d + CORT 8w	i. g.	*a, b*
Zhang et al. 2019b	CUMS	Male ICR	10	23–26/NR	Sal for 21d + CUMS 6 w	Flu (15 mg/kg) for 21d + CUMS 6w	BA (60 mg/kg/d) for 21d + CUMS 6w	i. g.	*a, c, d*
Zhang et al. 2019c	CRS	Male BALB/c	10	18–20/6	NR + CUMS 5w	Flu (25 mg/kg/d) for 14d + CUMS 5w	Api (60 mg/kg/d) for 14d + CUMS 5w	i. g.	*c, d*
Zhong et al. 2019	CUMS	Male C57BL/6	8	NR/ 7 to 8	Sal for 21d + CUMS 6w	Flu (20 mg/kg/d) for 21d + CUMS 6w	BA (50 mg/kg/d) for 21d + CUMS 6w	i. g.	*a, b, c, d, e, f, h*
Zhao et al. 2020	CORT	Female C57BL/6J	10	18–22/6 to 8	Sal for 42d + CORT (40 mg/kg/d) 6w	ES (10 mg/kg) for 21d + CORT (40 mg/kg/d) 6w	BA (60 mg/kg/d) for 21d + CORT (40 mg/kg/d) 6w	i. g.	*a, b, c, g*
Jia et al. 2021	CUMS	Male ICR	10	24 ± 2/7	Sal for 21d + CUMS 6w	Flu (10 mg/kg/d) for 21d + CUMS 6w	BA (50 mg/kg/d)for 21d + CUMS 6w	i. g.	*a, c*
Xiao et al. 2021	CUMS	Male ICR	9	20–25/7 to 8	NR+ CUMS 6w	Flu (10 mg/kg) for 21d + CUMS 6w	BA (100 mg/kg/d) for 21d + CUMS 6w	i. g.	*a, c*
Alghamdi et al. 2022	CMS	NR Albino	8	20–25/10 to 14	Sal for 21d + CUMS 3w	Imipramine (15 mg/kg/d) for 21d + CMS 3w	Api (50 mg/kg/d) for 21d + CMS 3w	i. g.	*a, c, g*
Al-Yamani et al. 2022	NR	Either sex Albino	6	20–25/ NR	NR	Flu (30 mg/kg) for three times	Api (50 mg/kg/d) for three times	i. g.	*a*
Liu et al. 2022a	LPS	Male C57BL/6	6	NR/NR	Sal + LPS (5 mg/kg) once	NR	Ba (3 mg/kg) once + LPS (5 mg/kg) once	i. p.	*a, b, e, f, h, i*
Li et al. 2022	LPS	Male C57BL/6	8	20 ± 2/8	0.5% CMC-Na + LPS (1 mg/kg) 3d	NR	Lut (50 mg/kg) 3d + LPS (1 mg/kg) 3d	i. g.	*b, c, e, h*
Lu et al. 2022	CUMS	Male C57BL/6N	9	18–20/6	Sal for 28d + CUMS 10w	Flu (10 mg/kg/d) for 28d + CUMS 10w	BA (10 mg/kg/d) for 28d + CUMS 10w	i. g.	*b, c*
Wang et al. 2023	CORT	Male ICR	8	26 ± 2/7	NR + CORT (40 mg/kg/d) 6w	Flu (10 mg/kg) for 21d + CORT (40 mg/kg/d) 6w	BA (50 mg/kg) for 21d + CORT (40 mg/kg/d) 6w	i. g.	*a, b, c*
Jin et al. 2023	CUMS	Male C57BL/6N	9	16–20/NR	Sal for 21d + CUMS 6w	Flu (20 mg/kg) for 21d + CUMS 6w	BA (20 mg/kg/d) for 21d + CUMS 6w	i. g.	*a, c*
Ma et al. 2023	CUMS	Male ICR	10	25–30/NR	water or 0.4% DMSO + CUMS 6w	Flu (20 mg/kg/d) for 21d + CUMS 6w	BA (60 mg/kg/d) for 21d + CUMS 6w	i. g.	*a, c*

Api: Apigenin; Ba: Baicalein; BA: baicalin; CMC-Na: sodium carboxymethyl cellulose; CMS: chronic mild stress; CORT: corticosterone; CRS: chronic restraint stress; CUMS: chronic unpredictable mild stress; DMSO: dimethylsulfoxide; Escitalopram: ES; Flu: fluoxetine; LPS: Lipopolysaccharide; Lut: Luteolin; Reb: Reboxetine; Saline: Sal; Vit: Vitexin; NR: not reported; i. g.: intragastric injection; i. p.:intraperitoneal injection; d: days; w: weeks; n: number.

*a*: tail suspension test (TST), *b*: forced swimming test (FST), *c*: sucrose preference test (SPT), *d*: open field test (OFT), *e*: IL-1β, *f*: TNF-α, *g*: serum corticosterone levels, *h*: IL-6, *i*: NF-kb, *j*: brain-derived neurotrophic factor (BDNF).

### Quality assessment of the included studies

**Figure 2.**
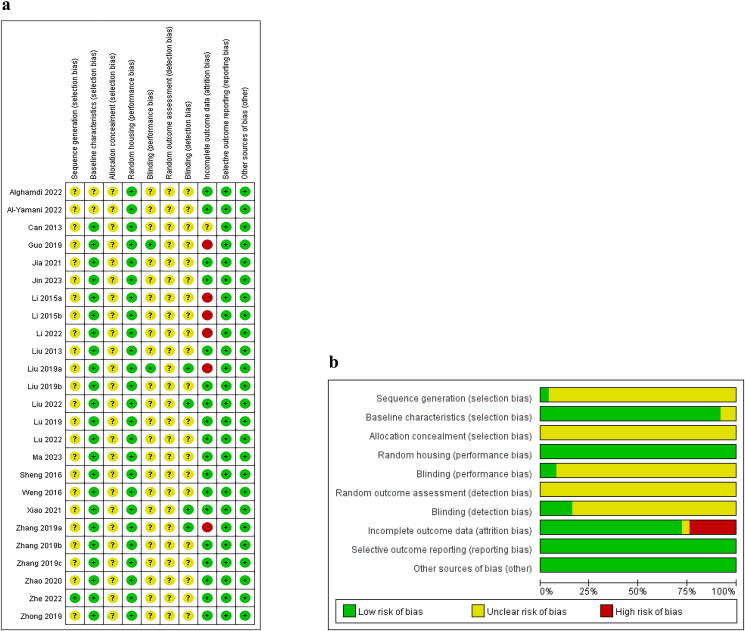
Risk of bias assessment table. (a) Risk of bias summary. (b) Risk of bias graph.

In the included literature, only one study described the specific randomization process in detail, while the rest mentioned ‘random’ methods only when allocating the animals (Wang et al. 2023). Twenty-three studies provided detailed descriptions of animal characteristics, ensuring similarity between groups. All studies were rated as unclear for allocation concealment due to the lack of detailed clarification regarding whether the animals were adequately concealed. Based on the descriptions of the experimental animals, the included studies were considered to be at low risk for issues with animal placement randomization. However, all were assessed as having an unclear risk in evaluating randomized outcomes. Experimental performers were blinded in two studies (Guo et al. 2019; Liu et al. 2019). No study described the assessment of outcomes in a randomized manner. Additionally, only four studies detailed the specific method of blinding used to evaluate outcomes (Liu et al. 2019; Zhang et al. 2019a; Xiao et al. 2021; Liu et al. 2022a). Due to missing data on the number of animals, six studies demonstrated a high risk of attrition bias (Li et al. 2015a; 2015b; Guo et al. 2019; Zhang et al. 2019a; Liu et al. 2019b; Li et al. 2022). The SYRCLE assessment of the risk of bias is shown in Figure 2.

### Outcome measures

#### Behavioral tests

**Figure 3.**
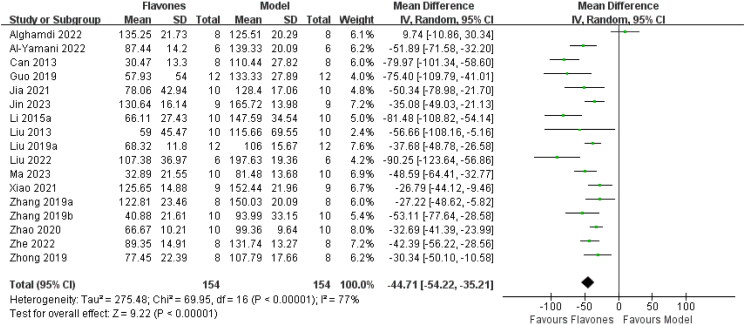
Forest plot for the effect of flavones on the tail suspension test.

Seventeen studies involving 308 mice reported on the TST (Can et al. 2013; Liu et al. 2013; Li et al. 2015a; Guo et al. 2019; Liu et al. 2019; Zhong et al. 2019; Zhang et al. 2019a; 2019b; Zhao et al. 2020; Jia et al. 2021; Xiao et al. 2021; Alghamdi et al. 2022; Al-Yamani et al. 2022; Liu et al. 2022a; Jin et al. 2023; Ma et al. 2023; Wang et al. 2023). The results of the meta-analysis indicated that flavones significantly reduced the immobility time in TST compared with the model group [MD = −44.71 (95% CI: −54.22, −35.21, *I^2^* = 77%; Figure 3)].

**Figure 4.**
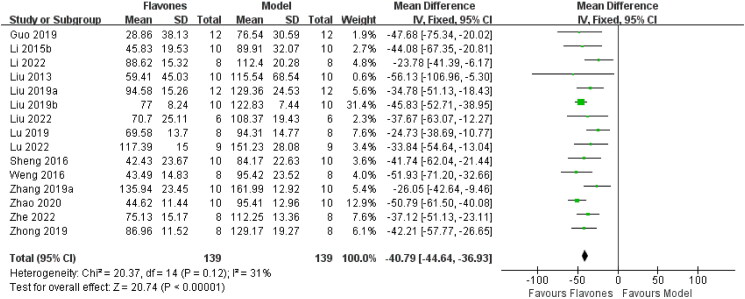
Forest plot for the effect of flavones on the forced swimming test.

A total of 15 studies, including (Liu et al. 2013; Li et al. 2015b; Sheng et al. 2016; Weng et al. 2016; Guo et al. 2019; Liu et al. 2019, 2019; Lu et al. 2019; Zhong et al. 2019; Zhang et al. 2019a; Zhao et al. 2020; Li et al. 2022; Lu et al. 2022; Liu et al. 2022a; Wang et al. 2023), involving 278 mice, reported on the FST. The pooled results indicated that flavones significantly reduced the immobility time in the FST compared to the model group [MD = −40.79 (95% CI: −44.64, −36.93, *I^2^* = 31%; Figure 4)].

**Figure 5.**
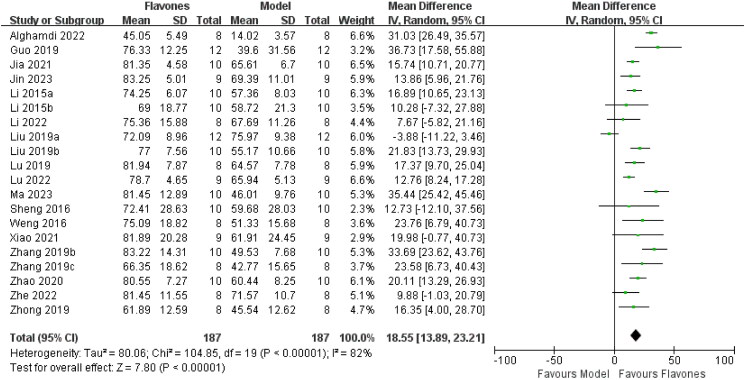
Forest plot for the effect of flavones on the sucrose preference test.

Twenty studies (Li et al. 2015a, 2015b; Sheng et al. 2016; Weng et al. 2016; Guo et al. 2019; Liu et al. 2019, 2019; Lu et al. 2019; Zhong et al. 2019; Zhang et al. 2019b, 2019c; Zhao et al. 2020; Jia et al. 2021; Xiao et al. 2021; Alghamdi et al. 2022; Li et al. 2022; Lu et al. 2022; Jin et al. 2023; Ma et al. 2023; Wang et al. 2023), including 374 mice assessed SPT. The meta-analysis revealed that, compared with the model group, flavones significantly increased the sucrose preference rate in an animal model of depression [MD = 18.55 (95% CI: 13.89, 23.21, *I^2^* = 82%; Figure 5)].

**Figure 6.**
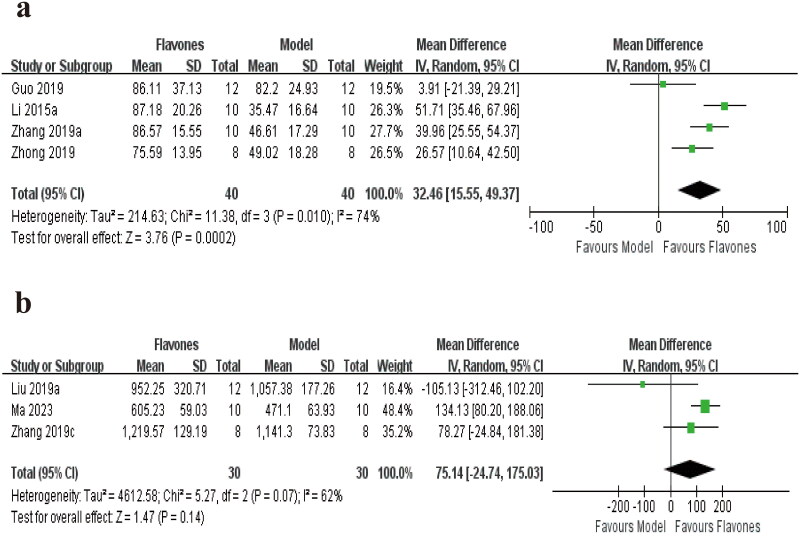
Forest plot for the effect of flavones on the (a) crossing number and (b) distance traveled.

Four studies involving 80 mice have reported the crossing number (Li et al. 2015a; Guo et al. 2019; Zhong et al. 2019; Zhang et al. 2019a). The pooled effects indicate that the administration of flavones was associated with a significant difference when compared to the model group [MD = 32.46 (95% CI: 15.55, 49.37, *I^2^* = 74%; Figure 6a)]; Additionally, three studies recorded the distance travelled (Liu et al. 2019; Zhang et al. 2019c; Ma et al. 2023), and the meta-analysis showed no significant difference in the distance travelled between the flavones group and the model group [MD = 75.14 (95% CI: −24.74, 175.03, *I^2^* = 62%; Figure 6b)].

#### Inflammatory cytokinesl

Three studies reported IL-1β levels in the blood (Liu et al. 2019; Zhong et al. 2019; Liu et al. 2022a); the results indicated a significant difference in reducing IL-1β levels between the flavone and model groups [MD = −17.74 (95% CI: −25.27, −10.2, *I^2^* = 0%; Appendix Files Figure S1)]. Three studies assessed the IL-1β levels in the hippocampus (pg/mg) (Guo et al. 2019; Zhong et al. 2019; Li et al. 2022), and the meta-analysis showed that there was no significant difference in reducing IL-1β levels between flavones and model groups [MD = −14.81 (95% CI: −37.79, 8.16, *I^2^* = 94%; Appendix Files Figure S2)]. Two studies presented IL-1β levels in the hippocampus (pg/ml) (Li et al. 2015a; Liu et al. 2019). The pooled effects showed a significant difference in reducing IL-1β levels between flavones and model groups [MD = −31.63 (95% CI: −48.02, −15.23, *I^2^* = 82%; Appendix Files Figure S3)].

Three studies presented the result of IL-6 levels in the blood (Liu et al. 2019; Zhong et al. 2019; Liu et al. 2022a); the findings indicated that there was no significant difference in reducing IL-6 levels between the flavone group and the model group [MD = −287.9 (95% CI: −607.32, 31.53, *I^2^* = 95%; Appendix Files Figure S4)]. A meta-analysis of three studies (Guo et al. 2019; Zhong et al. 2019; Li et al. 2022) revealed a significant difference in the reduction of IL-6 levels in the hippocampus between the flavone group and the model group [MD = −43.68 (95% CI: −80.05, −7.31, *I^2^* = 87%; Appendix Files Figure S5)].

Effect sizes for TNF-α levels were pooled from three studies (Liu et al. 2019; Zhong et al. 2019; Liu et al. 2022a). The pooled results showed that flavones significantly decreased TNF-α levels in the blood compared with those in the model group [MD = −22.23 (95% CI: −28.5, −15.95, *I^2^* = 37%; Appendix Files Figure S6)]. Two comparisons reported TNF-α levels in the hippocampus (pg/mg) (Guo et al. 2019; Zhong et al. 2019). The pooled effects showed no significant difference between the flavones and model groups [MD = −145.19 (95% CI: −379.98, 89.6, *I^2^* = 96%; Appendix Files Figure S7)]. Two comparisons reported the TNF-α levels in the hippocampus (pg/ml) (Li et al. 2015a; Liu et al. 2019), and the pooled results indicated that the administration of flavones was associated with a significant difference compared to the model group [MD = −41.27 (95% CI: −49.57, −32.97, *I^2^* = 34%; Appendix Files Figure S8)].

Concerning the effect on NF-κB levels in the prefrontal cortex (hippocampus), two studies reported this outcome (Li et al. 2015a; Liu et al. 2019). The pooled result showed that flavones significantly decreased NF-κB levels compared to the model group [MD = −0.23 (95% CI: −0.3, −0.16, *I^2^* = 0%; Appendix Files Figure S9)].

#### Other indexes

A total of three studies reported CORT levels (Li et al. 2015b; Weng et al. 2016; Zhao et al. 2020), and the results demonstrated that there was no significant difference between flavones and the model group [MD = −30.9 (95% CI: −80.39, 18.6, *I^2^* = 96%; Appendix Files Figure S10)].

Meta-analysis of three studies reported BDNF levels (Li et al. 2015b; Weng et al. 2016; Lu et al. 2019), and the results revealed that flavones can increase the BDNF levels in comparison with those in the model group [MD = 0.65 (95% CI: 0.41, 0.9, *I^2^* = 78%; Appendix Files Figure S11)].

#### Flavones compared with positive group

Compared with positive group, no significant difference was found between the positive group and the flavones group in reducing TST time [MD = 5.56 (95% CI: −1.83, 12.94, *I^2^* = 64%; Appendix Files Figure S12)], FST time [MD = −0.55 (95% CI: −9.38, 8.27, *I^2^* = 78%; Appendix Files Figure S13)], distance travelled [MD = 3.16 (95% CI: −91.76, 98.07, *I^2^* = 53%; Appendix Files Figure S14)], crossing number [MD = 3.42 (95% CI: −10.84, 17.68, *I^2^* = 51%; Appendix Files Figure S15)], IL-1β in blood level (pg/ml) [MD = 6.5 (95% CI: −1.49, 14.49, *I^2^* = 0%; Appendix Files Figure S16)], IL-1β in hippocampus level (pg/mg) [MD = 1.5 (95% CI: −11.26, 14.27, *I^2^* = 75%; Appendix Files Figure S17)], IL-1β in hippocampus level (pg/ml) [MD = 0.14 (95% CI: −22.07, 22.36, *I^2^* = 67%; Appendix Files Figure S18)], IL-6 in hippocampus level (pg/mg) [MD = 0.59 (95% CI: −12.5, 13.68, *I^2^* = 0%; Appendix Files Figure S19)], TNF-α in blood level (pg/ml) [MD = 6.46 (95% CI: 0.32, 12.61, *I^2^* = 16%; Appendix Files Figure S20)], TNF-α in hippocampus level (pg/mg) [MD = 25.71 (95% CI: −27.9, 79.32, *I^2^* = 70%; Appendix Files Figure S21)], TNF-α in hippocampus level (pg/ml) [MD = −3.88 (95% CI: −18.92, 11.17, *I^2^* = 79%; Appendix Files Figure S22)], NF-kb in blood level (pg/ml) [MD = 0.07 (95% CI: −0.06, 0.2, *I^2^* = 80%; Appendix Files Figure S23)] and BDNF levels [MD = 0.05 (95% CI: −0.08, 0.19, *I^2^* = 34%; Appendix Files Figure S24)].

The pooled result showed that flavones increase IL-6 in blood level (pg/ml) [MD = 9.82 (95% CI: 3.56, 16.09, *I^2^* = 8%; Appendix Files Figure S25)], sucrose preference rate [MD = 2.47 (95% CI: 0.84, 4.1, *I^2^* = 32%; Appendix Files Figure S26)] and CORT levels [MD = −10.48 (95% CI: −19.43, −1.54, *I^2^* = 0%; Appendix Files Figure S27)] in an animal model of depression.

### Publication bias

Egger’s test indicated that there was no publication bias with the TST, FST, and SPT in the model group (*p* = 0.078, *p* = 0.329, *p* = 0.980) and the positive group (*p* = 0.28, *p* = 0.225, *p* = 0.195). Since fewer than ten studies were included, a publication bias assessment was not conducted for the other outcomes.

### Subgroup and sensitivity analyses

Considering the high heterogeneity in the meta-analyses, we conducted further subgroup analyses based on the type of flavone (vitexin, apigenin, baicalin, baicalein, luteolin), the method of administration (intragastrically vs. intraperitoneally) and the dose. The flavone dosage was categorized into three groups: low (less than 50 mg/kg/day), medium (greater than 50 mg/kg/day and less than 100 mg/kg/day), and high (greater than 100 mg/kg/day). For the TST, FST, and SPT, the subgroup analyses did not reveal significant differences between the flavone group and the model or positive groups based on either the type of flavone, dose and the method of administration.

**Figure 7.**
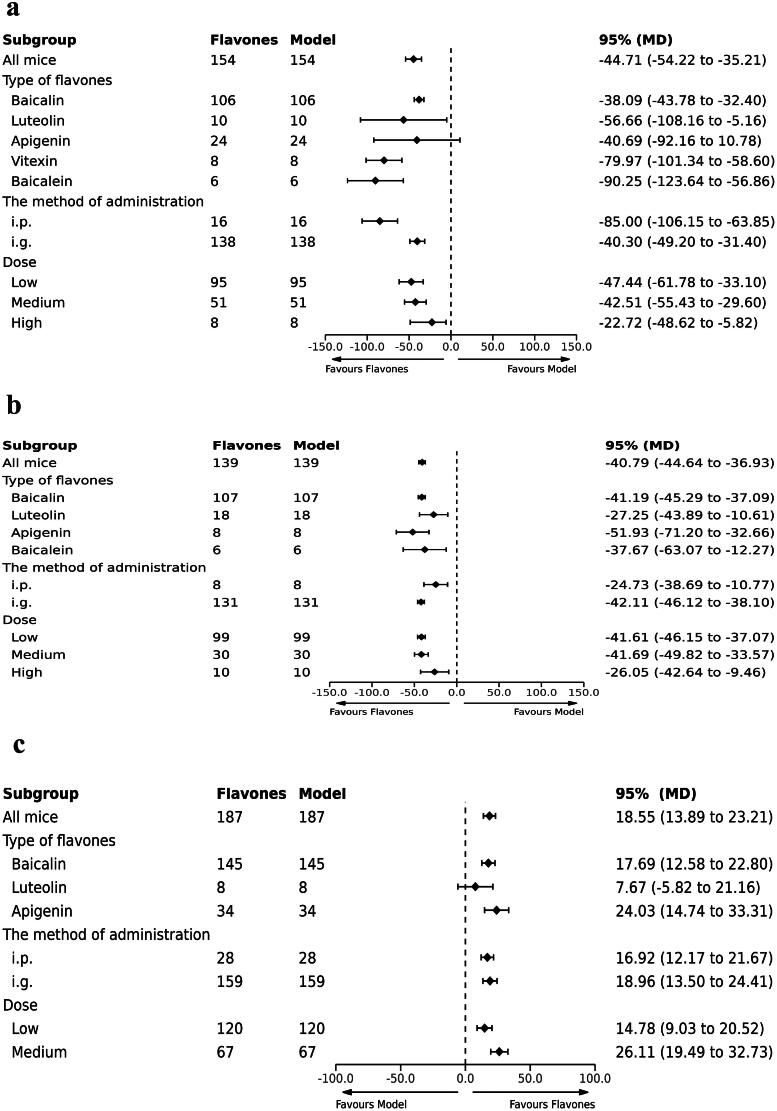
Subgroup analysis of pooled estimates of behavioral tests on the (a) tail suspension test, (b) forced swimming test and (c) sucrose preference test.

In assessing the antidepressant effects of flavones compared to the model group, a subgroup analysis of different types of flavones was conducted for the TST. It was observed that the heterogeneity decreased in the baicalin group. The heterogeneity of the TST significantly decreased from 77% to 26%, suggesting that the type of flavone and the dosage were the primary contributors to heterogeneity. However, the heterogeneity of the apigenin group remained high. Similarly, sources of heterogeneity in the SPT results were analyzed, and even after subgroup analysis, the heterogeneity remained high (Figure 7).

On the antidepressant effects of flavones compared with the positive group in meta-analyses, for the TST, the heterogeneity decreased sharply from 63% to 0%, and the results were unaffected by the exclusion of the study by Jin et al. (2023). For the FST, subgroup analyses of the outcomes showed a high degree of heterogeneity (Figure S28).

When we conducted a sensitivity analysis by excluding studies that did not explicitly indicate the missing data, the results of the TST, FST, and SPT remained relatively unchanged, suggesting that the results were stable (Figures S29–S34).

### Quality of evidence

Utilizing the GRADE approach, we assessed the quality of evidence for the reported outcomes. In the model group, the evidence quality was classified as moderate (*n* = 4) and low (*n* = 12). The findings from the model group are summarized in Table 2, while those from the positive group are detailed in Appendix Files, Tab. S3.

**Table 2.** Assessment of quality and summarizing the findings using the GRADE approach.

Certainty assessment							No of animals	Effect		Quality of the evidence (GRADE)
Outcomes	No of studies	Risk of bias	Inconsistency	Indirectness	Imprecision	Other considerations	Flavones /Model	Relative (95% CI)	Absolute	
TST	17	Serious^a^	Not serious^b^	Not serious	Not serious	None	154/154	–	MD 44.71 lower (54.22 lower to 35.21 lower)	⨁⨁⨁◯ Moderate
FST	15	Serious^a^	Not serious^b^	Not serious	Not serious	None	139/139	–	MD 40.79 lower (44.64 lower to 36.93 lower)	⨁⨁⨁◯ Moderate
SPT	20	Serious^a^	Serious^c^	Not serious	Not serious	None	187/187	–	MD 18.55 higher (13.89 higher to 23.21 higher)	⨁⨁◯◯ Low
OFT (crossing number)	4	Serious^a^	Serious^c^	Not serious	Not serious	None	40/40	–	MD 32.46 higher (15.55 higher to 49.37 higher)	⨁⨁◯◯ Low
OFT (distance traveled)	3	Serious^a^	Serious^c^	Not serious	Not serious	None	30/30	**-**	MD 75.14 higher (24.74 lower to 175.03 higher)	⨁⨁◯◯ Low
IL-1β levels in blood (pg/ml)	3	Serious^a^	Not serious	Not serious	Not serious	None	20/20	**-**	MD 17.74 lower (25.27 lower to 10.2 lower)	⨁⨁⨁◯ Moderate
IL-1β levels in the hippocampus (pg/mg)	3	Serious^a^	Serious^c^	Not serious	Not serious	None	24/24	**-**	MD 14.81 lower (37.79 lower to 8.16 higher)	⨁⨁◯◯ Low
IL-1β levels in the hippocampus (pg/ml)	2	Serious^a^	Serious^c^	Not serious	Not serious	None	12/12	**-**	MD 31.63 lower (48.02 lower to 15.23 lower)	⨁⨁◯◯ Low
IL-6 levels in the blood (pg/ml)	3	Serious^a^	Serious^c^	Not serious	Not serious	None	20/20	**-**	MD 287.9 lower (607.32 lower to 31.53 higher)	⨁⨁◯◯ Low
IL-6 levels in the hippocampus (pg/mg)	3	Serious^a^	Serious^c^	Not serious	Not serious	None	24/24	**-**	MD 43.68 lower (80.05 lower to 7.31 lower)	⨁⨁◯◯ Low
TNF-α levels in the blood (pg/ml)	3	Serious^a^	Not serious	Not serious	Not serious	None	20/20	**-**	MD 22.23 lower (28.5 lower to 15.95 lower)	⨁⨁◯◯ Low
TNF-α levels in the hippocampus (pg/mg)	2	Serious^a^	Serious^c^	Not serious	Not serious	None	16/16	**-**	MD 145.19 lower (379.98 lower to 89.6 higher)	⨁⨁◯◯ Low
TNF-α levels in the hippocampus (pg/ml)	2	Serious^a^	Not serious	Not serious	Not serious	None	12/12	**-**	MD 41.27 lower (49.57 lower to 32.97 lower)	⨁⨁◯◯ Low
NF-κB levels in the prefrontal cortex	2	Serious^a^	Not serious	Not serious	Not serious	None	9/9	**-**	MD 0.23 lower (0.3 lower to 0.16 lower)	⨁⨁⨁◯ Moderate
CORT levels	3	Serious^a^	Serious^c^	Not serious	Not serious	None	28/28	**-**	MD 30.9 lower (80.39 lower to 18.6 higher)	⨁⨁◯◯ Low
BDNF levels	3	Serious^a^	Serious^c^	Not serious	Not serious	None	21/21	**-**	MD 0.65 higher (0.41 higher to 0.9 higher)	⨁⨁◯◯ Low

*Note:* High quality: we are very confident that the true effect lies close to that of the estimate of the effect. Moderate quality: we are moderately confident in the effect estimate: the true effect is likely to be close to the estimate of the effect, but there is a possibility that it is substantially different. Low quality: our confidence in the effect estimate is limited: the true effect may be substantially different from the estimate of the effect. Very low quality: we have very little confidence in the effect estimate: the true effect is likely to be substantially different from the estimate of effect.

CI: confidence interval; MD: mean difference.

^a^ The included studies were significantly biased with respect to randomization methods, allocation concealment, blind method, and incomplete outcome dat.

^b^ There is no serious inconsistency since the sources of heterogeneity were identified.

^c^ Heterogeneity (*I^2^* > 50%, *p* < 0.05) was found.

### Trial sequential analysis

**Figure 8.**
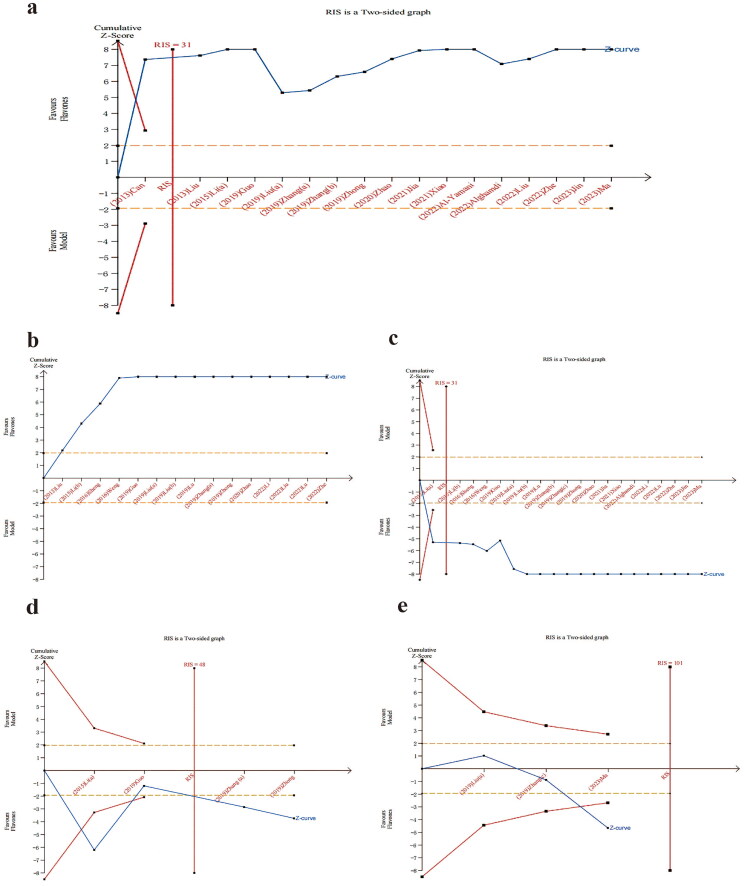
Trial sequential analysis for (a) tail suspension test, (b) forced swimming test, (c) sucrose preference test, (d) OFT (crossing number) and (e) OFT (distance traveled).

The result of TSA on TST, FST, SPT and OFT (crossing number) showed that the cumulative z-value surpassed both the conventional boundary and the TSA threshold, thereby achieving the Required Information Size (RIS). However, for the distance traveled, the cumulative z-value failed to reach the RIS, though it did cross the TSA boundary value. Despite not achieving the anticipated sample size, clinical efficacy has been established. These findings demonstrate the effectiveness of flavones in treating depression in comparison with the model group (Figure 8).

The results of the TSA for TST, SPT, and OFT (crossing number) indicated that the cumulative Z-value surpassed both the conventional boundary and the TSA threshold, thereby achieving the Required Information Size (RIS). For the FST, the software reported that the first information fraction exceeded 100% of the RIS, suggesting that the available sample size was sufficient to provide statistically reliable conclusions. However, for the distance traveled, the cumulative Z-value did not reach the RIS, although it did surpass the TSA boundary value. These findings demonstrate the effectiveness of flavones in treating depression in comparison with the model group (Figure 8).

The findings indicate that additional studies are still required to confirm the comparative analysis of flavones and positive control drugs in the treatment of depression (Appendix Files Figure S35).

## Discussion

### Efficacy of flavones

Globally, depression is one of the leading causes of disability and mobility impairment (Herrman et al. 2019). Depression may be caused by multiple factors, and its complex mechanism makes prevention and treatment challenging (Harmer et al. 2017). Consequently, it is essential to identify more efficacious treatments. Natural compounds have been investigated for their antidepressant properties. Polyphenols like flavones, naturally occurring polyphenols, have been extensively researched for their pharmacological properties, particularly their antidepressant properties. Studies with rodents have shown that they reverse depressive behaviors. Several previous *in vivo* preclinical studies in animal models and human patients had reported the therapeutic potential of flavones on depression (Al-Yamani et al. 2022; Zhang et al. 2023). For instance, A flavonoid-rich fraction from *Scutellaria baicalensis*, an herb used in traditional Chinese medicine, may provide antidepressant effects (Wang and Gao 2024).

This meta-analysis included 458 mice. Based on the results of this meta-analysis, flavones are effective in a variety of experimental models. We observed that, compared to the model group, flavones demonstrated a significant reduction in immobility time in both FST and TST, increased SPT, elevated locomotor activity, and decreased levels of pro-inflammatory cytokines, namely IL-1β, IL-6, TNF-α, and NF-κB. These findings collectively suggest that flavones have the potential to significantly ameliorate depressive behavior, with their anti-inflammatory effects emerging as a plausible explanation for the observed protective outcomes.

In our meta-analysis, we observed a pronounced degree of heterogeneity in the primary outcomes, including TST, FST, and SPT, a phenomenon anticipated in animal experimental studies.This heterogeneity has a substantial impact on the robustness and reliability of our analytical results. The results of our subgroup analysis indicate that this high level of heterogeneity could potentially be attributed to variations in the types of flavones used, the doses of administered drugs and the design of animal experiments. The selection of flavones is of paramount importance in this context. Baicalin and its aglycone, baicalein, are flavonoid compounds isolated from the roots of *Scutellaria baicalensis*. Apigenin is a naturally derived compound that can be found abundantly in fruits and vegetables, while luteolin is widely distributed among plant species. Vitexin, classified as a flavone C-glucoside, is present in nutraceuticals and food items and has garnered attention owing to its potential antidepressant-like effects. These compounds demonstrate prominent biological activities, including anti-inflammatory, neuroprotective properties and antioxidant (Can et al. 2013; Guo et al. 2019; Zhang et al. 2019c; Li et al. 2022).

According to subgroup analyses, the effects of baicalin, apigenin, luteolin, and vitexin treatments on depressive-like behaviors, as measured by FST and SPT, were not statistically significant compared to the control group. Nonetheless, the collective findings suggest that the administration of flavones demonstrated greater efficacy in alleviating depressive-like symptoms relative to the control group. Conversely, concerning TST, the observed difference in the effectiveness of apigenin in mitigating depressive behaviors in mice compared to the control group did not reach statistical significance. This outcome could potentially be attributed to the limited inclusion of studies. Notably, in two studies involving baicalin, data loss was documented in the reports of TST, FST, and SPT.

We conducted a subgroup analysis to assess the impact of different flavone doses. Our findings indicated that the administration of flavones at low doses did not result in notable improvements in behavioral performance compared to the outcomes observed in the groups receiving medium and high doses relative to the control group. Furthermore, our analysis demonstrated that, compared to the positive control group, interventions involving medium doses of flavones were more effective in improving behavioral outcomes than those using low doses.

However, since the animal models included in the study were all mouse models, we cannot determine whether the findings from these models can be reliably extrapolated to other animal models or clinical studies. Additionally, our study presents inconclusive results when comparing the efficacy of flavone compounds with positive control medications for treating depression, primarily due to the limited sample size of the included trials. Therefore, further evidence from clinical trials is needed to evaluate the efficacy of flavones, and several high-quality studies with larger sample sizes are necessary to confirm our findings. Moreover, analyzing the factors contributing to heterogeneity poses challenges. Due to the limited sample size, accurately identifying the precise causes of the observed high heterogeneity proved complex.

### Mechanisms

To date, the mechanism of action of antidepressants is recognized as multifaceted, encompassing increased neurotransmitter levels, receptor activity modulation, alterations in neuroplasticity, exertion of anti-inflammatory effects, and stimulation of neurotrophic factors (Liu et al. 2015; Peng et al. 2015). Each element contributes distinctively to the therapeutic impact of antidepressants, with the potential to operate independently or synergistically to alleviate depressive symptoms. Current research on the antidepressant mechanisms of flavones primarily involves: (1) Elevation of the monoamine, including norepinephrine (NA), 5-HT, and dopamine (DA) pathways, are implicated in depression’s pathophysiology (Dean and Keshavan 2017). Vitexin’s antidepressant efficacy hinges on increased catecholamine levels and interactions with 5-HT_1A_, dopaminergic D_1_, D_2_, and D_3_ receptors (Can et al. 2013). Additionally, apigenin’s antidepressant effects involve α-adrenergic, dopaminergic, and 5-HT_3_ serotonergic receptors (Al-Yamani et al. 2022); (2) Anti-inflammatory effects, increasing evidence associates inflammation with depression’s etiology, inducing depressive behaviors (Dean and Keshavan 2017). Baicalin mitigates neuroinflammation and depressive-like symptoms by inhibiting toll-like receptor 4 *via* the phosphatidylinositol 3-kinase (PI3K)/protein kinase B (AKT)/forkhead box protein O1 (FoxO1) pathway, suppressing high mobility group box protein 1 (HMGB1)/toll-like receptor 4 (TLR4)/NF-κB pathways, and regulating N-methyl-D aspartate receptor (NMDAR)/subtype 2B (NR2B)- extracellular regulated kinase (ERK)1/2 to curb inflammation and oxidative stress (Zhong et al. 2019; Liu et al. 2022a). Apigenin reduces nitric-oxide synthase (iNOS) and cyclooxygenase-2 (COX-2) expressions by activating NF-kB (Li et al. 2015a), while luteolin promotes anti-neuroinflammatory and antidepressant effects by upregulating A20 and Nrf2 (Li et al. 2022); (3) Regulation of the hypothalamic-pituitary-adrenal (HPA) axis, baicalin modulates the HPA axis by reducing serum corticosterone and increasing 11β-hydroxysteroid dehydrogenase-2 (11β-HSD2) and BDNF in the hippocampus (Li et al. 2015b). It also remodels HPA axis feedback by normalizing glucocorticoid receptor (GR) nuclear translocation (Zhang et al. 2019a) and activates the PI3K/AKT/glycogen synthase kinase 3β (GSK3β)/β-catenin pathway (Zhao et al. 2020). (4) Promoting neurogenesis, baicalin has been demonstrated to reverse CUMS-induced reductions in protein kinase B (p-Akt), forkhead box transcription factor (FOXG1), and fibroblast growth factor (FGF2) (Zhang et al. 2019b). BDNF, crucial for neuronal survival, is found to be reduced in the hippocampus of individuals with depression (Peng et al. 2015; Colucci-D’Amato et al. 2020). Additionally, apigenin has been shown to modulate levels of corticosterone and increase BDNF in the hippocampus (Sheng et al. 2016; Weng et al. 2016). Baicalin’s antidepressant effects involve BDNF/ERK/cAMP response element binding (CREB), BDNF/tropomyosin-related kinase B (TrkB)/ras-related C3 botulinum toxin substrate (Rac)/LIM kinase (LIMK) pathways (Lu et al. 2019; Jia et al. 2021), mammalian target of rapamycin (mTOR)/glycogen synthase kinase 3β (GSK3β) (Wang et al. 2023) and Wnt/β-catenin signaling pathway (Xiao et al. 2021); (5) Inhibition of oxidative stress. Various preclinical and clinical trials have shown that oxidative stress may be a potentially important parameter for depression (Alghamdi et al. 2022; Liu et al. 2023). For instance, compared to the control group, apigenin alleviated CMS-induced emotional behaviours in mice by increasing the glutathione level and significantly decreasing malondialdehyde (MDA) and catalase levels in the brain (Alghamdi et al. 2022). Additionally, luteolin diminished MDA levels, while the glutathione peroxidase (GSH) and superoxide dismutase (SOD) levels were decreased in the hippocampus of CUMS exposed mice and also reduced depression-like behaviours (Liu et al. 2013); (6) Inhibition of neuronal apoptosis, another molecular mechanism that maintains cellular homeostasis, has recently been shown to be associated with the physiology of depression (Wang et al. 2021). Based on several animal depression models, baicalin attenuated the behaviour of depression hippocampal neuronal apoptosis by regulating the silent information regulator sirtuin 1 (SIRT1)/polymerase-1 (PARP1) signaling pathway (Ma et al. 2023) and inducing the expression of calbindin-D28K (Liu et al. 2019); (7) Improvement of mitochondrial dysfunction. Jin et al. discovered that baicalin might enhance nip-like protein (NIX)-mediated mitophagy through direct binding to adenosine monophosphate-activated protein kinase (AMPK) and activating the downstream step of the proliferator-activated receptor-gamma coactivator-1 (PGC-1) signaling pathway (Jin et al. 2023). This mechanism may contribute to baicalin’s antidepressant effects by upregulating genes related to enzymes in glycolysis and the tricarboxylic acid cycle, thereby improving mitochondrial function and increasing adenosine triphosphate (ATP) levels in the brain. Additionally, findings suggest that apigenin may facilitate autophagy *via* the AMPK/mammalian target of the rapamycin (mTOR) pathway, inducing antidepressive effects in mice subjected to chronic restraint stress (Zhang et al. 2019c). (8) Regulation of the Gut–Brain Axis, emerging preclinical studies have demonstrated that the changes in the composition and function of gut microbiota are associated with the development and progression of depression, primarily *via* the regulation of the gut-brain axis (Liu et al. 2022b). Research demonstrating that quercetin may exert antidepressant effects by restoring gut microbiota balance thorough its targeted modulation of specific bacterial populations, including *Romboutsia*, *Turicibacter*, *Faecalibaculum*, and *Bifidobacterium*. Additionally, it may exert its effects by influencing brain metabolism (Li et al. 2024).

### Strengths and limitations

This meta-analysis has several strengths. First, compared with previous research, our study is the first preclinical meta-analysis to assess the impact of flavones in combating depression in animal models. As a result of our findings, we can provide practical applications for transforming animal data from the laboratory to the bedside using evidence-based methods. Secondly, we performed a comparative analysis of the antidepressant effects of flavone compounds against both positive drugs and the model group. To further investigate the antidepressant effectiveness, we conducted subgroup analyses with different flavones and doses. Thirdly, we assessed the evidence using the GRADE system for evaluating the quality of evidence.

This meta-analysis has several limitations. Firstly, only a small number of studies with small sample sizes were included. Secondly, the acquisition of original data was incomplete, with some data points obtained using the WebPlotDigitizer program. As a result, there may be errors that could affect the accuracy of the results, potentially leading to measurement bias. Thirdly, the literature quality assessment using the risk of bias tool revealed a lack of high-quality studies, particularly in terms of randomization, blinding methods, and handling of missing data, which may contribute to selection bias. Biased or imprecise results and without rigorous evaluation, some preclinical animal studies supported by clinical research led to costly clinical research and withdrawal from the market afterward. The effects of drugs on human patients with depression can be better predicted in human models of depression that are of higher quality. To provide high-quality evidence, future research should focus on improving experimental methods, modeling approaches, and reporting animal experiments. Therefore, some conclusions in the present study should be interpreted with caution. As a result of this result, we should increase the sample size and standardize the experimental design in future studies to make the results more reliable.

## Conclusions

In summary, our findings indicate that flavones can significantly ameliorate depression-like behaviors and enhance neurological function in animals exhibiting depressive symptoms. However, there is still a need for larger animal models and clinical trials to validate the results. Therefore, additional high-quality preclinical trials and clinical studies are essential before flavones can be applied in human treatments.

## Supplementary Material

Supplementary_Material.pdf

## Data Availability

All relevant data supporting the findings of this study are included in the main text and supplementary materials. Additional data will be made available on request.
