# Supplementary material for: Antidepressant activity of flavones from traditional Chinese medicine: a meta-analysis
Source: Pharm Biol. 2025 Feb 25;63(1):156–69. doi: 10.1080/13880209.2025.2467374 (PMC11864034; doi:10.1080/13880209.2025.2467374)

Antidepressant activity of flavones from traditional Chinese medicine: a meta-analysis

Qing Wang [a,b], Youyuan Lu [a,c], Xue Mi [b], Caiyan Yang [b], Wei Ma [b], Changbo Xia [d], Hanqing Wang [a,c,e,*]

[a] *College of Pharmacy, Ningxia Medical University, Yinchuan, China*
[b] *Department of Pharmacy, The First People's Hospital of Yinchuan, Yinchuan, China*
[c] *Ningxia Regional Characteristic Traditional Chinese Medicine Collaborative Innovation Center Co-constructed by the Province and Ministry, Ningxia Engineering and Technology Research Center for Modernization of Regional Characteristic Traditional Chinese Medicine, Ningxia Medical University, Yinchuan, China*
[d] *Department of Pharmacy,Central's Hospital of Xinxiang, Xinxiang, China*
[e] *Key Laboratory of Ningxia Minority Medicine Modernization, Ministry of Education, Ningxia Medical University, Yinchuan, China*

*** Correspondence:**
Professor Hanqing Wang
Address: School of Pharmacy, Ningxia Medical University, Yinchuan 750004, China
Tel: +86-18909590509
Fax: +86-0951-6980192
E-mail: wwwhhq@163.com

Supplementary Material

Table S1. PRISMA (Preferred Reporting Items for Systematic Review and Meta-Analysis) checklist

Section and Topic	Item #	Checklist item	Location where item is reported	
**TITLE**				
Title	1	Identify the report as a systematic review.	Page 2	
**ABSTRACT**				
Abstract	2	See the PRISMA 2020 for Abstracts checklist.	Page 4	
**INTRODUCTION**				
Rationale	3	Describe the rationale for the review in the context of existing knowledge.	Page 3	
Objectives	4	Provide an explicit statement of the objective(s) or question(s) the review addresses.	Page 3	
**METHODS**				
Eligibility criteria	5	Specify the inclusion and exclusion criteria for the review and how studies were grouped for the syntheses.	Page 3	
Informatio n sources	6	Specify all databases, registers, websites, organisations, reference lists and other sources searched or consulted to identify studies. Specify the date when each source was last searched or consulted.	Page 3	
Search strategy	7	Present the full search strategies for all databases, registers and websites, including any filters and limits used.	Page 4	
Selection process	8	Specify the methods used to decide whether a study met the inclusion criteria of the review, including how many reviewers screened each record and each report retrieved, whether they worked independently, and if applicable, details of automation tools used in the process.	Page 4	
Data collection process	9	Specify the methods used to collect data from reports, including how many reviewers collected data from each report, whether they worked independently, any processes for obtaining or confirming data from study investigators, and if applicable, details of automation tools used in the process.	Page 5	
Data items	10a	List and define all outcomes for which data were sought. Specify whether all results that were compatible with each outcome domain in each study were sought (e.g. for all measures, time points, analyses), and if not, the	Page 5	

Section and Topic	Item #	Checklist item	Location where item is reported	
		methods used to decide which results to collect.		
	10b	List and define all other variables for which data were sought (e.g. participant and intervention characteristics, funding sources). Describe any assumptions made about any missing or unclear information.	Page 5	
Study risk of bias assessment	11	Specify the methods used to assess risk of bias in the included studies, including details of the tool(s) used, how many reviewers assessed each study and whether they worked independently, and if applicable, details of automation tools used in the process.	Page 4-5	
Effect measures	12	Specify for each outcome the effect measure(s) (e.g. risk ratio, mean difference) used in the synthesis or presentation of results.	Page 5	
Synthesis methods	13a	Describe the processes used to decide which studies were eligible for each synthesis (e.g. tabulating the study intervention characteristics and comparing against the planned groups for each synthesis (item #5)).	Page 5	
	13b	Describe any methods required to prepare the data for presentation or synthesis, such as handling of missing summary statistics, or data conversions.	Page 5	
	13c	Describe any methods used to tabulate or visually display results of individual studies and syntheses.	Page 5	
	13d	Describe any methods used to synthesize results and provide a rationale for the choice(s). If meta-analysis was performed, describe the model(s), method(s) to identify the presence and extent of statistical heterogeneity, and software package(s) used.	Page 5	
	13e	Describe any methods used to explore possible causes of heterogeneity among study results (e.g. subgroup analysis, meta-regression).	Page 5	
	13f	Describe any sensitivity analyses conducted to assess robustness of the synthesized results.	Page 5	
Reporting bias assessment	14	Describe any methods used to assess risk of bias due to missing results in a synthesis (arising from reporting biases).	Page 5	
Certainty assessment	15	Describe any methods used to assess certainty (or confidence) in the body of evidence for an outcome.	Page 5	
**RESULTS**				
Study	16a	Describe the results of the search and selection process, from the number of records identified in the search to	Page 5	

Section and Topic	Item #	Checklist item	Location where item is reported	
selection		the number of studies included in the review, ideally using a flow diagram.		
	16b	Cite studies that might appear to meet the inclusion criteria, but which were excluded, and explain why they were excluded.	Page 5	
Study characteristics	17	Cite each included study and present its characteristics.	Page 5	
Risk of bias in studies	18	Present assessments of risk of bias for each included study.	Page 6	
Results of individual studies	19	For all outcomes, present, for each study: (a) summary statistics for each group (where appropriate) and (b) an effect estimate and its precision (e.g. confidence/credible interval), ideally using structured tables or plots.	Page 6-8	
Results of syntheses	20a	For each synthesis, briefly summarise the characteristics and risk of bias among contributing studies.	Page 6-8	
	20b	Present results of all statistical syntheses conducted. If meta-analysis was done, present for each the summary estimate and its precision (e.g. confidence/credible interval) and measures of statistical heterogeneity. If comparing groups, describe the direction of the effect.	Page 6-8	
	20c	Present results of all investigations of possible causes of heterogeneity among study results.	Page 8	
	20d	Present results of all sensitivity analyses conducted to assess the robustness of the synthesized results.	Page 8-9	
Reporting biases	21	Present assessments of risk of bias due to missing results (arising from reporting biases) for each synthesis assessed.	Page 8-9	
Certainty of evidence	22	Present assessments of certainty (or confidence) in the body of evidence for each outcome assessed.	Page 8-9	
DISCUSSION				
Discussion	23a	Provide a general interpretation of the results in the context of other evidence.	Page 9-12	
	23b	Discuss any limitations of the evidence included in the review.	Page 9-12	
	23c	Discuss any limitations of the review processes used.	Page 9-12	
	23d	Discuss implications of the results for practice, policy, and future research.	Page 9-12	

Section and Topic	Item #	Checklist item	Location where item is reported	
**OTHER INFORMATION**				
Registration and protocol	24a	Provide registration information for the review, including register name and registration number, or state that the review was not registered.	Page 4	
	24b	Indicate where the review protocol can be accessed, or state that a protocol was not prepared.	Page 4	
	24c	Describe and explain any amendments to information provided at registration or in the protocol.	N/A	
Support	25	Describe sources of financial or non-financial support for the review, and the role of the funders or sponsors in the review.	Page 12	
Competing interests	26	Declare any competing interests of review authors.	Page 13	
Availability of data, code and other materials	27	Report which of the following are publicly available and where they can be found: template data collection forms; data extracted from included studies; data used for all analyses; analytic code; any other materials used in the review.	The data supporting the findings of this study are available within the article and its supplementary materials.	

From: Page MJ, McKenzie JE, Bossuyt PM, Boutron I, Hoffmann TC, Mulrow CD, et al. The PRISMA 2020 statement: an updated guideline for reporting systematic reviews. BMJ 20a21;372:n71. doi: 10.1136/bmj.n7

Table S2. Search strategies through electronic databases.

Databases	Detailed search strategies
PubMed	**#1.** (depression or Depressive Disorder[MeSH Terms]) OR (Depressive Symptoms[Title/Abstract] OR Depressive Symptom[Title/Abstract] OR Emotional Depression[Title/Abstract] OR Depressive Disorders[Title/Abstract] OR Depressive Neuroses[Title/Abstract] OR Depressive Neurosis[Title/Abstract] OR Endogenous Depression*[Title/Abstract] OR Depressive Syndrome*[Title/Abstract] OR Neurotic Depression*[Title/Abstract] OR Melancholia*[Title/Abstract] OR Unipolar Depression[Title/Abstract] OR Unipolar Depressions[Title/Abstract] OR antidepressant[Title/Abstract] OR antidepressant-like effect[Title/Abstract] OR Anti-Depressive[Title/Abstract] OR Depressive-Like[Title/Abstract])<br>**#2.** (Models, Animal or Animal Experimentation [MeSH Terms]) OR (animal Model*[Title/Abstract] OR Laboratory Animal Model*[Title/Abstract] OR Experimental Animal Model*[Title/Abstract] OR Animal Research[Title/Abstract] OR Animal Experimental Use*[Title/Abstract] OR Animal Experiment*[Title/Abstract] OR animal[Title/Abstract] OR mice[Title/Abstract] OR Mouse[Title/Abstract] OR experimental[Title/Abstract])<br>**#3.** ((apigenin or luteolin[MeSH Terms]) OR (Baicalin[Supplementary Concept] OR Vitexin[Supplementary Concept] OR baicalein[Supplementary Concept])) OR (Luteoline[Title/Abstract])<br>**#4.** #1 AND #2 AND #3
Embase	**#1.** 'depression'/exp<br>**#2.** depressive:ti,ab,kw AND symptoms:ti,ab,kw OR (depressive:ti,ab,kw AND symptom:ti,ab,kw) OR (emotional:ti,ab,kw AND depression:ti,ab,kw) OR (depressive:ti,ab,kw AND disorders:ti,ab,kw) OR (depressive:ti,ab,kw AND neuroses:ti,ab,kw) OR (depressive:ti,ab,kw AND neurosis:ti,ab,kw) OR (endogenous:ti,ab,kw AND depression*:ti,ab,kw) OR (depressive:ti,ab,kw AND syndrome*:ti,ab,kw) OR (neurotic:ti,ab,kw AND depression*:ti,ab,kw) OR melancholia*:ti,ab,kw OR (unipolar:ti,ab,kw AND depression:ti,ab,kw) OR (unipolar:ti,ab,kw AND depressions:ti,ab,kw) OR antidepressant:ti,ab,kw OR ('antidepressant like':ti,ab,kw AND effect:ti,ab,kw) OR 'anti depressive':ti,ab,kw OR 'depressive like':ti,ab,kw<br>**#3.** #1 OR #2<br>**#4.** 'animal model'/exp<br>**#5.** 'animal experiment'/exp<br>**#6.** 'mouse'/exp<br>**#7.** animal:ti,ab,kw AND model*:ti,ab,kw OR (laboratory:ti,ab,kw AND animal:ti,ab,kw AND model*:ti,ab,kw) OR (experimental:ti,ab,kw AND animal:ti,ab,kw AND model*:ti,ab,kw) OR (animal:ti,ab,kw AND research:ti,ab,kw) OR (animal:ti,ab,kw AND experimental:ti,ab,kw) OR (animal:ti,ab,kw AND experiment*:ti,ab,kw) OR animal:ti,ab,kw OR mice:ti,ab,kw OR mouse:ti,ab,kw OR experimental:ti,ab,kw<br>**#8.** #4 OR #5 OR #6 OR #7<br>**#9.** 'apigenin'/exp<br>**#10.** 'luteolin'/exp<br>**#11.** 'baicalin'/exp<br>**#12.** 'vitexin'/exp<br>**#13.** 'baicalein'/exp<br>**#14.** #9 OR #10 OR #11 OR #12 OR #13<br>**#15.** #3 AND #8 AND #14
Web of Science	**#1.** TS=(depression or Depressive Disorder or Depressive Symptoms or Depressive Symptom or Emotional Depression or Depressive Disorders or Depressive Neuroses or Depressive Neurosis or Endogenous Depression* or Depressive Syndrome* or Neurotic Depression* or Melancholia* or Unipolar Depression or Unipolar Depressions or antidepressant or antidepressant-like effect or Anti-Depressive or Depressive-Like)<br>**#2.** TS=(Models, Animal or Animal Experimentation or Laboratory Animal Model* or Experimental Animal Model* or Animal Research or Animal Experimental Use* or Animal Experiment* or animal or mice or experimental)<br>**#3.** TS=(apigenin or luteolin or Baicalin or Vitexin or baicalein or Luteoline)<br>**#4.** #1 AND #1 AND #3

Sinomed, China National Knowledge Infrastructure, China Science and Technology Journal Database, and Wanfang Data	（抑郁症 or 抑郁性神经症 or 神经性抑郁 or 抑郁性障碍 or 抑郁障碍 or 抑郁障碍症 or 抑郁综合征 or 单相抑郁症 or 忧郁症 or 抗抑郁）and（动物 or 鼠）and（芹菜素 or 木犀草素 or 黄芩苷 or 牡荆素 or 黄芩素）and 体内试验

Table S3. Assessment of quality and summarizing the findings using the GRADE approach (compared with the positive group).

Outcomes	Certainty assessment						No of animals	Effect		Quality of the evidence (GRADE)
	No of studies	Risk of bias	Inconsistency	Indirectness	Imprecision	Other considerations	Flavones /Positive	Relative (95% CI)	Absolute	
TST	15	serious[a]	not serious[b]	not serious	not serious	none	138/138	-	MD 5.56 higher (1.83 lower to 12.94 higher)	⊕⊕⊕◯ Moderate
FST	12	serious[a]	serious[c]	not serious	not serious	none	115/115	-	MD 0.55 lower (9.38 lower to 8.27 higher)	⊕⊕◯◯ Low
SPT	19	serious[a]	not serious	not serious	not serious	none	179/179	-	MD 2.47 higher (0.84 higher to 4.1 higher)	⊕⊕⊕◯ Moderate
OFT (crossing number)	4	serious[a]	serious[c]	not serious	not serious	none	40/40	-	MD 3.42 higher (10.84 lower to 17.68 higher)	⊕⊕◯◯ Low
OFT (distance traveled)	3	serious[a]	serious[c]	not serious	not serious	none	30/30	-	MD 3.16 higher (91.76 lower to 98.07 higher)	⊕⊕◯◯ Low
IL-1β levels in blood (pg/ml)	2	serious[a]	not serious	not serious	not serious	none	14/14	-	MD 6.5 higher (1.49 lower to 14.49 higher)	⊕⊕⊕◯ Moderate
IL-1β levels in the hippocampus (pg/mg)	2	serious[a]	serious[c]	not serious	not serious	none	16/16	-	MD 1.5 higher (11.26 lower to 14.27 higher)	⊕⊕◯◯ Low
IL-1β levels in the hippocampus (pg/ml)	2	serious[a]	serious[c]	not serious	not serious	none	12/12	-	MD 0.14 higher (22.07 lower to 22.36 higher)	⊕⊕◯◯ Low
IL-6 levels in the hippocampus (pg/mg)	2	serious[a]	not serious	not serious	not serious	none	16/16	-	MD 0.59 higher (12.5 lower to 13.68 higher)	⊕⊕⊕◯ Moderate
IL-6 levels in the blood (pg/ml)	2	serious[a]	not serious	not serious	not serious	none	14/14	-	MD 9.82 higher (3.56 higher to 16.09 higher)	⊕⊕⊕◯ Moderate
TNF-α levels in the blood (pg/ml)	2	serious[a]	not serious	not serious	not serious	none	14/14	-	MD 6.46 higher	⊕⊕⊕◯ Moderate

									(0.32 higher to 12.61 higher)	
TNF-α levels in the hippocampus (pg/mg)	2	serious[a]	serious[c]	not serious	not serious	none	16/16	-	MD 25.71 higher (27.9 lower to 79.32 higher)	⊕⊕◯◯ Low
TNF-α levels in the hippocampus (pg/ml)	2	serious[a]	serious[c]	not serious	not serious	none	12/12	-	MD 3.88 lower (18.92 lower to 11.17 higher)	⊕⊕◯◯ Low
NF-κB levels in the prefrontal cortex	2	serious[a]	serious[c]	not serious	not serious	none	9/9	-	MD 0.07 higher (0.06 lower to 0.2 higher)	⊕⊕◯◯ Low
CORT levels	3	serious[a]	not serious[b]	not serious	not serious	none	28/28	-	MD 10.48 lower (19.43 lower to 1.54 lower)	⊕⊕⊕◯ Moderate
BDNF levels	3	serious[a]	not serious[b]	not serious	not serious	none	21/21	-	MD 0.05 higher (0.08 lower to 0.19 higher)	⊕⊕⊕◯ Moderate

Note: High quality: we are very confident that the true effect lies close to that of the estimate of the effect. Moderate quality: we are moderately confident in the effect estimate: the true effect is likely to be close to the estimate of the effect, but there is a possibility that it is substantially different. Low quality: our confidence in the effect estimate is limited: the true effect may be substantially different from the estimate of the effect.Very low quality: we have very little confidence in the effect estimate: the true effect is likely to be substantially different from the estimate of effect.

CI: confidence interval; MD: mean difference.

[a]The includedstudies were significantly biased with respect to randomization methods, allocation concealment, blind method, and incomplete outcome dat.

[b]There is no serious inconsistency since the sources of heterogeneity were identified.

[c]Heterogeneity ($I^2 > 50\%$, $p < 0.05$) was found.

Figure S1. Forest for the IL-1β levels in the blood.

Study or Subgroup	Flavones Mean	Flavones SD	Flavones Total	Model Mean	Model SD	Model Total	Weight	Mean Difference IV, Random, 95% CI
Liu 2019a	39.64	9.6	6	62.09	11.27	6	40.4%	-22.45 [-34.30, -10.60]
Liu 2022	7.45	2.43	6	19.41	14.5	6	41.0%	-11.96 [-23.72, -0.20]
Zhong 2019	39.49	13.22	8	59.71	21.48	8	18.6%	-20.22 [-37.70, -2.74]
**Total (95% CI)**			**20**			**20**	**100.0%**	**-17.74 [-25.27, -10.20]**

Heterogeneity: Tau² = 0.00; Chi² = 1.61, df = 2 (P = 0.45); I² = 0%
Test for overall effect: Z = 4.61 (P < 0.00001)

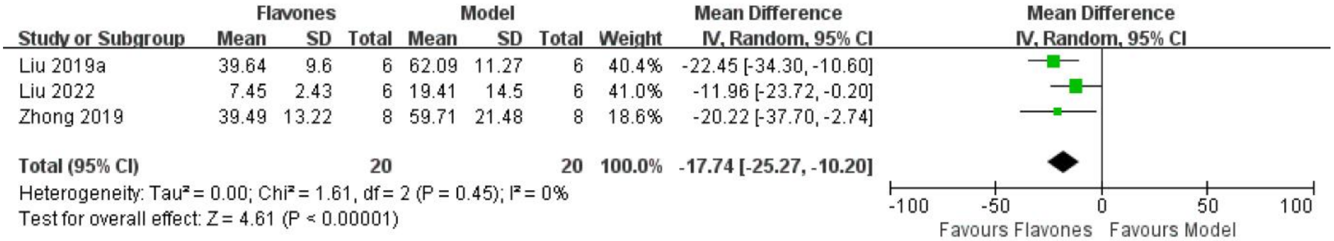

Figure S2. Forest for the IL-1β levels in the hippocampus (pg/mg).

Study or Subgroup	Flavones Mean	Flavones SD	Flavones Total	Model Mean	Model SD	Model Total	Weight	Mean Difference IV, Random, 95% CI
Guo 2019	25.14	7.22	8	36	5.24	8	34.7%	-10.86 [-17.04, -4.68]
Li 2022	54.39	11.97	8	47.21	10.87	8	32.8%	7.18 [-4.02, 18.38]
Zhong 2019	32.67	8.72	8	73.98	15.08	8	32.4%	-41.31 [-53.38, -29.24]
**Total (95% CI)**			**24**			**24**	**100.0%**	**-14.81 [-37.79, 8.16]**

Heterogeneity: Tau² = 385.76; Chi² = 34.00, df = 2 (P < 0.00001); I² = 94%
Test for overall effect: Z = 1.26 (P = 0.21)

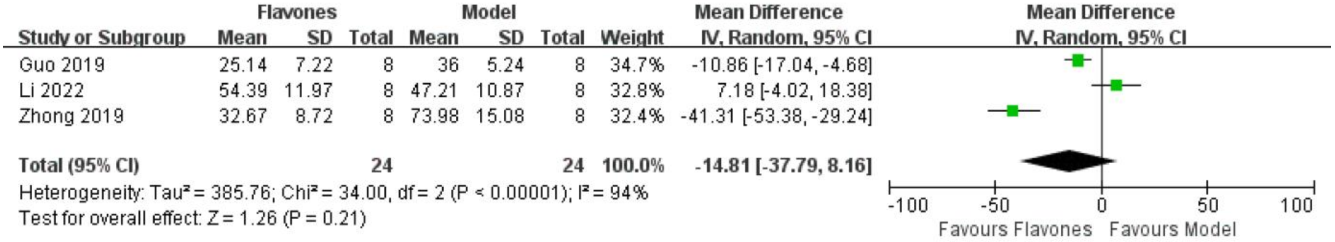

Figure S3. Forest for the IL-1β levels in the hippocampus (pg/ml).

Study or Subgroup	Flavones Mean	Flavones SD	Flavones Total	Model Mean	Model SD	Model Total	Weight	Mean Difference IV, Random, 95% CI
Li 2015a	66.47	9.33	6	106.29	6.93	6	51.0%	-39.82 [-49.12, -30.52]
Liu 2019a	39.52	9.04	6	62.61	9.36	6	49.0%	-23.09 [-33.50, -12.68]
**Total (95% CI)**			**12**			**12**	**100.0%**	**-31.63 [-48.02, -15.23]**

Heterogeneity: Tau² = 114.58; Chi² = 5.52, df = 1 (P = 0.02); I² = 82%
Test for overall effect: Z = 3.78 (P = 0.0002)

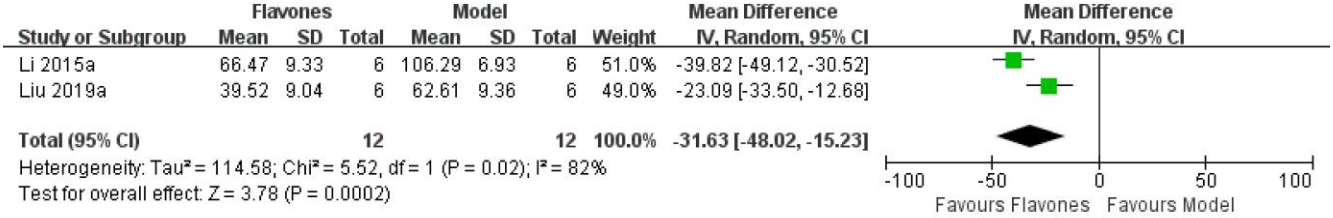

Figure S4. Forest for the IL-6 levels in the blood.

Study or Subgroup	Flavones Mean	Flavones SD	Flavones Total	Model Mean	Model SD	Model Total	Weight	Mean Difference IV, Random, 95% CI
Liu 2019a	184.62	54.81	6	613.52	205.6	6	32.3%	-428.90 [-599.16, -258.64]
Liu 2022	184.62	54.81	6	613.52	205.6	6	32.3%	-428.90 [-599.16, -258.64]
Zhong 2019	50.47	13.22	8	81.98	18.88	8	35.5%	-31.51 [-47.48, -15.54]
**Total (95% CI)**			**20**			**20**	**100.0%**	**-287.90 [-607.32, 31.53]**

Heterogeneity: Tau² = 74790.97; Chi² = 41.13, df = 2 (P < 0.00001); I² = 95%
Test for overall effect: Z = 1.77 (P = 0.08)

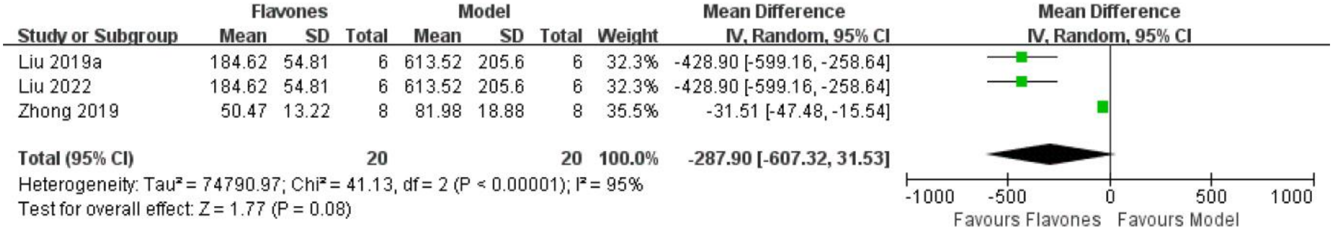

Figure S5. Forest for the IL-6 levels in the hippocampus.

Study or Subgroup	Flavones Mean	Flavones SD	Flavones Total	Model Mean	Model SD	Model Total	Weight	Mean Difference IV, Random, 95% CI
Guo 2019	277.95	25.64	8	404.43	75.87	8	21.4%	-126.48 [-181.98, -70.98]
Li 2022	69.38	22.27	8	99	12.74	8	38.6%	-29.62 [-47.40, -11.84]
Zhong 2019	34.1	16.16	8	47.19	11.15	8	40.1%	-13.09 [-26.69, 0.51]
**Total (95% CI)**			**24**			**24**	**100.0%**	**-43.68 [-80.05, -7.31]**

Heterogeneity: Tau² = 810.96; Chi² = 15.93, df = 2 (P = 0.0003); I² = 87%

Test for overall effect: Z = 2.35 (P = 0.02)

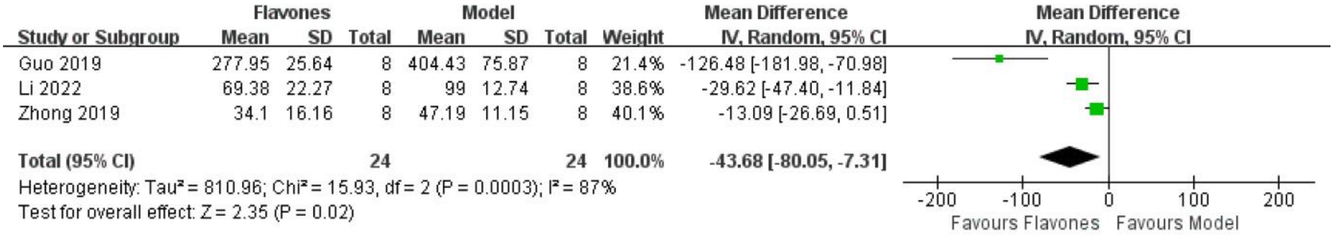

Figure S6. Forest for the TNF-α levels in the blood.

Study or Subgroup	Flavones Mean	Flavones SD	Flavones Total	Model Mean	Model SD	Model Total	Weight	Mean Difference IV, Fixed, 95% CI
Liu 2019a	61.54	7.33	6	82.9	8.77	6	47.1%	-21.36 [-30.51, -12.21]
Liu 2022	12.91	3.9	6	31.14	12.05	6	38.3%	-18.23 [-28.36, -8.10]
Zhong 2019	57.07	11.74	8	92.62	20.6	8	14.6%	-35.55 [-51.98, -19.12]
**Total (95% CI)**			**20**			**20**	**100.0%**	**-22.23 [-28.50, -15.95]**

Heterogeneity: Chi² = 3.16, df = 2 (P = 0.21); I² = 37%

Test for overall effect: Z = 6.94 (P < 0.00001)

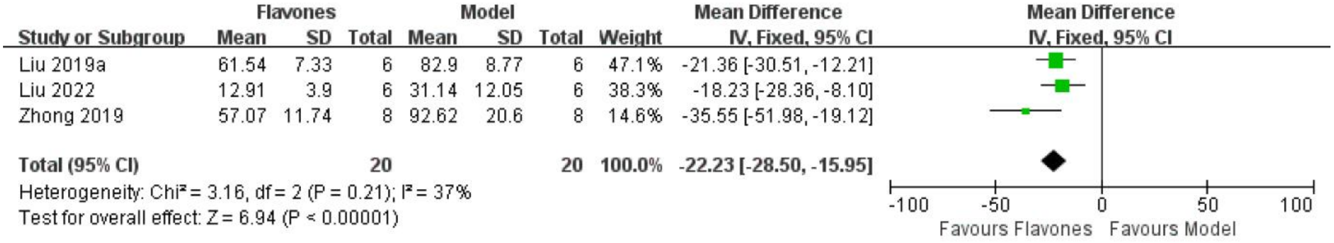

Figure S7. Forest for the TNF-α levels in the hippocampus (pg/mg) .

Study or Subgroup	Flavones Mean	Flavones SD	Flavones Total	Model Mean	Model SD	Model Total	Weight	Mean Difference IV, Random, 95% CI
Guo 2019	392.59	69.87	8	662.53	118.86	8	48.0%	-269.94 [-365.48, -174.40]
Zhong 2019	50.26	13.5	8	80.42	13.58	8	52.0%	-30.16 [-43.43, -16.89]
**Total (95% CI)**			**16**			**16**	**100.0%**	**-145.19 [-379.98, 89.60]**

Heterogeneity: Tau² = 27536.21; Chi² = 23.74, df = 1 (P < 0.00001); I² = 96%

Test for overall effect: Z = 1.21 (P = 0.23)

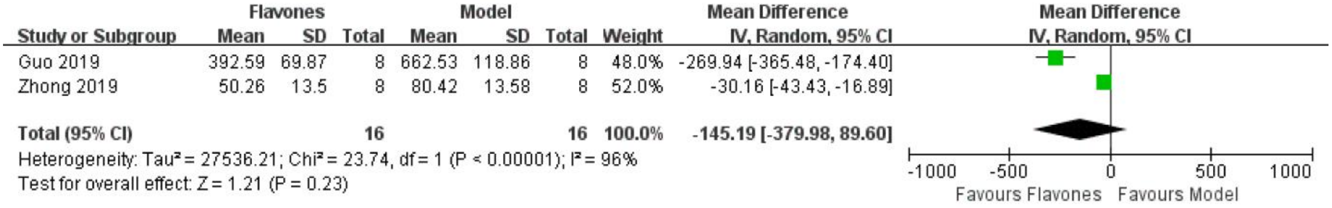

Figure S8. Forest for the TNF-α levels in the hippocampus.

Study or Subgroup	Flavones Mean	Flavones SD	Flavones Total	Model Mean	Model SD	Model Total	Weight	Mean Difference IV, Fixed, 95% CI
Li 2015a	56.2	6.39	6	103.94	15.26	6	39.3%	-47.74 [-60.98, -34.50]
Liu 2019a	54.98	8.5	6	92.06	10.24	6	60.7%	-37.08 [-47.73, -26.43]
**Total (95% CI)**			**12**			**12**	**100.0%**	**-41.27 [-49.57, -32.97]**

Heterogeneity: Chi² = 1.51, df = 1 (P = 0.22); I² = 34%

Test for overall effect: Z = 9.75 (P < 0.00001)

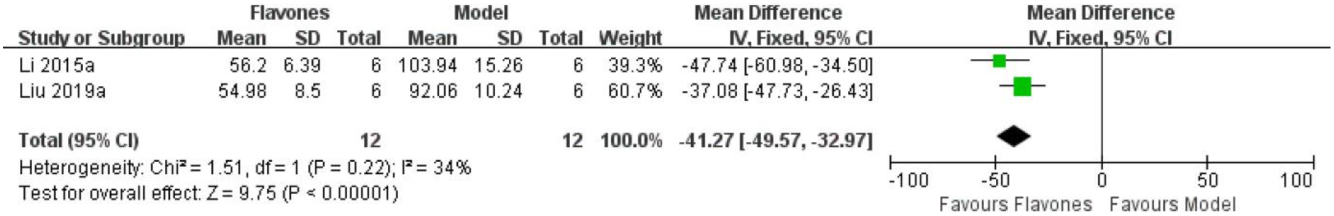

Figure S9. Forest for the NF-κB levels in the hippocampus.

Study or Subgroup	Flavones Mean	Flavones SD	Flavones Total	Model Mean	Model SD	Model Total	Weight	Mean Difference IV, Fixed, 95% CI
Li 2015a	0.38	0.05	3	0.62	0.05	3	66.7%	-0.24 [-0.32, -0.16]
Liu 2019a	0.53	0.1	6	0.74	0.1	6	33.3%	-0.21 [-0.32, -0.10]
**Total (95% CI)**			**9**			**9**	**100.0%**	**-0.23 [-0.30, -0.16]**

Heterogeneity: Chi² = 0.18, df = 1 (P = 0.67); I² = 0%
Test for overall effect: Z = 6.90 (P < 0.00001)

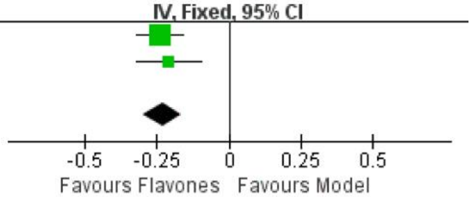

Figure S10. Forest for the CORT levels in the hippocampus.

Study or Subgroup	Flavones Mean	Flavones SD	Flavones Total	Model Mean	Model SD	Model Total	Weight	Mean Difference IV, Random, 95% CI
Li 2015b	43.83	19.53	10	71.96	24.3	10	33.1%	-28.13 [-47.45, -8.81]
Weng 2016	43.49	14.83	8	120.68	30.71	8	32.3%	-77.19 [-100.82, -53.56]
Zhao 2020	44.62	11.44	10	34.94	4.17	10	34.6%	9.68 [2.13, 17.23]
**Total (95% CI)**			**28**			**28**	**100.0%**	**-30.90 [-80.39, 18.60]**

Heterogeneity: Tau² = 1828.91; Chi² = 55.11, df = 2 (P < 0.00001); I² = 96%
Test for overall effect: Z = 1.22 (P = 0.22)

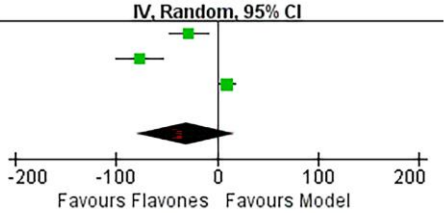

Figure S11. Forest for the BDNF levels.

Study or Subgroup	Flavones Mean	Flavones SD	Flavones Total	Model Mean	Model SD	Model Total	Weight	Mean Difference IV, Random, 95% CI
Li 2015b	0.97	0.13	5	0.49	0.02	5	42.0%	0.48 [0.36, 0.60]
Lu 2019	1.17	0.23	8	0.58	0.11	8	37.3%	0.59 [0.41, 0.77]
Weng 2016	1.46	0.4	8	0.35	0.42	8	20.7%	1.11 [0.71, 1.51]
**Total (95% CI)**			**21**			**21**	**100.0%**	**0.65 [0.41, 0.90]**

Heterogeneity: Tau² = 0.03; Chi² = 9.06, df = 2 (P = 0.01); I² = 78%
Test for overall effect: Z = 5.20 (P < 0.00001)

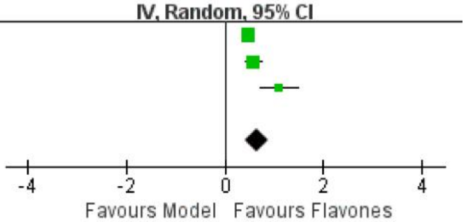

Figure S12. Forest plot for the effect of flavones on the tail suspension test (compared with the positive group).

Study or Subgroup	Flavones Mean	Flavones SD	Flavones Total	Positive Mean	Positive SD	Positive Total	Weight	Mean Difference IV, Random, 95% CI
Alghamdi 2022	135.25	21.73	8	125.51	20.29	8	6.1%	9.74 [-10.86, 30.34]
Al-Yamani 2022	87.44	14.26	6	77.44	19.94	6	6.4%	10.00 [-9.62, 29.62]
Can 2013	30.47	13.3	8	41.22	17.74	8	7.7%	-10.75 [-26.11, 4.61]
Guo 2019	57.93	54.6	12	52.77	59.55	12	2.1%	5.16 [-40.55, 50.87]
Jia 2021	78.06	42.94	10	71.17	29.64	10	3.6%	6.89 [-25.45, 39.23]
Jin 2023	130.64	16.14	9	84.89	18.63	9	7.4%	45.75 [29.65, 61.85]
Li 2015a	66.11	27.43	10	64.77	31.66	10	4.8%	1.34 [-24.62, 27.30]
Liu 2019a	68.32	11.8	12	64.92	14.5	12	9.3%	3.40 [-7.18, 13.98]
Ma 2023	32.89	21.55	10	31.68	20.29	10	6.7%	1.21 [-17.14, 19.56]
Xiao 2021	125.65	14.88	9	125.9	14.88	9	8.2%	-0.25 [-14.00, 13.50]
Zhang 2019a	122.81	23.46	8	113.88	18.06	8	6.1%	8.93 [-11.59, 29.45]
Zhang 2019b	40.88	21.61	10	39.17	18.36	10	7.0%	1.71 [-15.87, 19.29]
Zhao 2020	66.67	10.21	10	75.82	11.5	10	9.7%	-9.15 [-18.68, 0.38]
Zhe 2022	89.35	14.91	8	84.09	14.4	8	8.0%	5.26 [-9.10, 19.62]
Zhong 2019	77.45	22.39	8	66.71	11.46	8	7.0%	10.74 [-6.69, 28.17]
**Total (95% CI)**			**138**			**138**	**100.0%**	**5.56 [-1.83, 12.94]**

Heterogeneity: $Tau^2 = 123.55$; $Chi^2 = 38.73$, df = 14 (P = 0.0004); $I^2 = 64\%$

Test for overall effect: Z = 1.47 (P = 0.14)

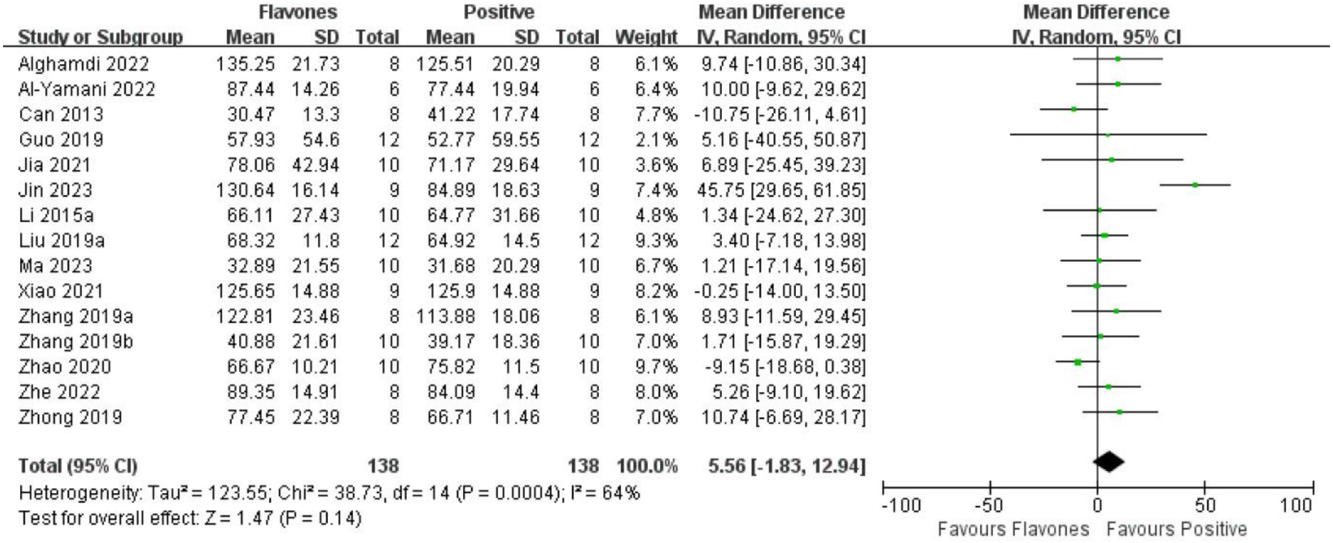

Figure S13. Forest plot for the effect of flavones on the forced swimming test (compared with the positive group).

Study or Subgroup	Flavones Mean	Flavones SD	Flavones Total	Positive Mean	Positive SD	Positive Total	Weight	Mean Difference IV, Random, 95% CI
Guo 2019	28.86	38.13	12	28.66	35.36	12	5.1%	0.20 [-29.22, 29.62]
Li 2015b	43.83	19.53	10	53.73	23.45	10	7.6%	-9.90 [-28.81, 9.01]
Liu 2019a	94.58	15.26	12	77.76	15.26	12	9.5%	16.82 [4.61, 29.03]
Liu 2019b	77	8.24	10	88.5	6.09	10	10.9%	-11.50 [-17.85, -5.15]
Lu 2019	69.58	13.7	8	89.96	11.72	8	9.4%	-20.38 [-32.87, -7.89]
Lu 2022	117.39	15	9	89.01	21.69	9	8.0%	28.38 [11.15, 45.61]
Sheng 2016	42.43	23.67	10	51.41	24.21	10	7.0%	-8.98 [-29.97, 12.01]
Weng 2016	43.49	14.83	8	44.8	22.24	8	7.7%	-1.31 [-19.83, 17.21]
Zhang 2019a	135.94	23.45	10	139.06	19.75	10	7.5%	-3.12 [-22.12, 15.88]
Zhao 2020	44.62	11.44	10	60.1	15.89	10	9.5%	-15.48 [-27.62, -3.34]
Zhe 2022	75.13	15.17	8	69.51	13.02	8	9.0%	5.62 [-8.23, 19.47]
Zhong 2019	86.96	11.52	8	70.92	17.69	8	8.8%	16.04 [1.41, 30.67]
**Total (95% CI)**			**115**			**115**	**100.0%**	**-0.55 [-9.38, 8.27]**

Heterogeneity: $Tau^2 = 175.22$; $Chi^2 = 49.95$, df = 11 (P < 0.00001); $I^2 = 78\%$

Test for overall effect: Z = 0.12 (P = 0.90)

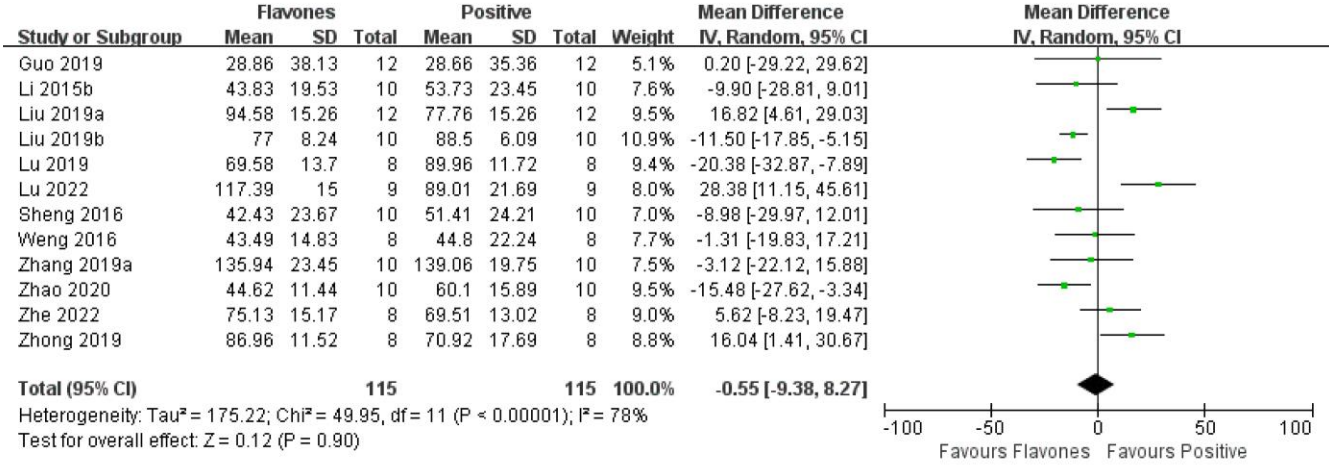

Figure S14. Forest plot for the effect of flavones on the distance traveled (compared with the positive group).

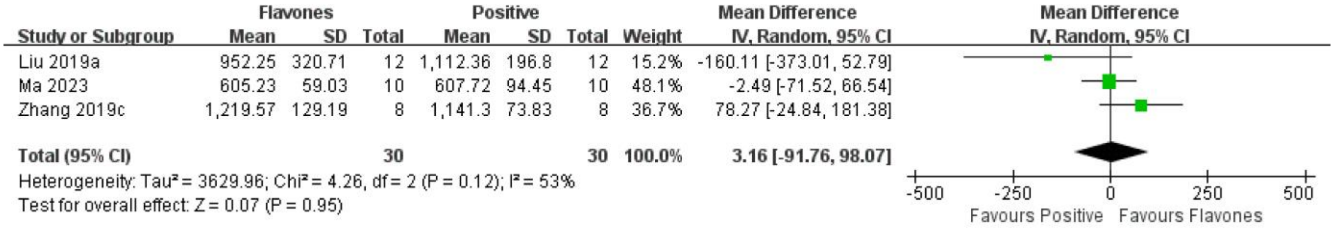

Study or Subgroup	Flavones Mean	Flavones SD	Flavones Total	Positive Mean	Positive SD	Positive Total	Weight	Mean Difference IV, Random, 95% CI
Liu 2019a	952.25	320.71	12	1,112.36	196.8	12	15.2%	-160.11 [-373.01, 52.79]
Ma 2023	605.23	59.03	10	607.72	94.45	10	48.1%	-2.49 [-71.52, 66.54]
Zhang 2019c	1,219.57	129.19	8	1,141.3	73.83	8	36.7%	78.27 [-24.84, 181.38]
**Total (95% CI)**			**30**			**30**	**100.0%**	**3.16 [-91.76, 98.07]**

Heterogeneity: Tau² = 3629.96; Chi² = 4.26, df = 2 (P = 0.12); I² = 53%
Test for overall effect: Z = 0.07 (P = 0.95)

Figure S15. Forest plot for the effect of flavones on the crossing number (compared with the positive group).

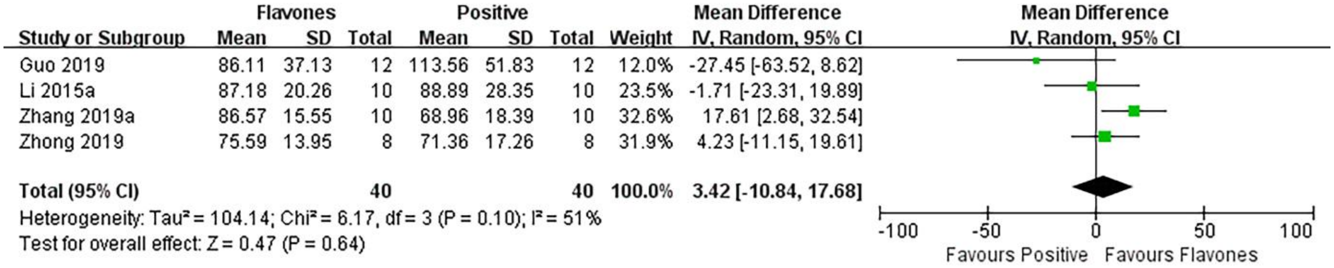

Study or Subgroup	Flavones Mean	Flavones SD	Flavones Total	Positive Mean	Positive SD	Positive Total	Weight	Mean Difference IV, Random, 95% CI
Guo 2019	86.11	37.13	12	113.56	51.83	12	12.0%	-27.45 [-63.52, 8.62]
Li 2015a	87.18	20.26	10	88.89	28.35	10	23.5%	-1.71 [-23.31, 19.89]
Zhang 2019a	86.57	15.55	10	68.96	18.39	10	32.6%	17.61 [2.68, 32.54]
Zhong 2019	75.59	13.95	8	71.36	17.26	8	31.9%	4.23 [-11.15, 19.61]
**Total (95% CI)**			**40**			**40**	**100.0%**	**3.42 [-10.84, 17.68]**

Heterogeneity: Tau² = 104.14; Chi² = 6.17, df = 3 (P = 0.10); I² = 51%
Test for overall effect: Z = 0.47 (P = 0.64)

Figure S16. Forest for the IL-1β levels in the blood (pg/ml) (compared with the positive group).

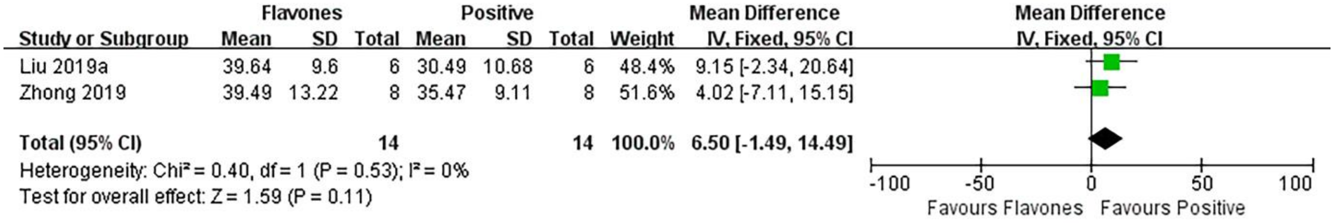

Study or Subgroup	Flavones Mean	Flavones SD	Flavones Total	Positive Mean	Positive SD	Positive Total	Weight	Mean Difference IV, Fixed, 95% CI
Liu 2019a	39.64	9.6	6	30.49	10.68	6	48.4%	9.15 [-2.34, 20.64]
Zhong 2019	39.49	13.22	8	35.47	9.11	8	51.6%	4.02 [-7.11, 15.15]
**Total (95% CI)**			**14**			**14**	**100.0%**	**6.50 [-1.49, 14.49]**

Heterogeneity: Chi² = 0.40, df = 1 (P = 0.53); I² = 0%
Test for overall effect: Z = 1.59 (P = 0.11)

Figure S17. Forest for the IL-1β levels in the hippocampus (pg/mg) (compared with the positive group).

	Flavones			Positive				Mean Difference
Study or Subgroup	Mean	SD	Total	Mean	SD	Total	Weight	IV, Random, 95% CI
Guo 2019	25.14	7.22	8	30.49	12.34	8	47.4%	-5.35 [-15.26, 4.56]
Zhong 2019	32.67	8.72	8	24.98	7.67	8	52.6%	7.69 [-0.36, 15.74]
**Total (95% CI)**			**16**			**16**	**100.0%**	**1.50 [-11.26, 14.27]**

Heterogeneity: Tau² = 63.82; Chi² = 4.01, df = 1 (P = 0.05); I² = 75%
Test for overall effect: Z = 0.23 (P = 0.82)

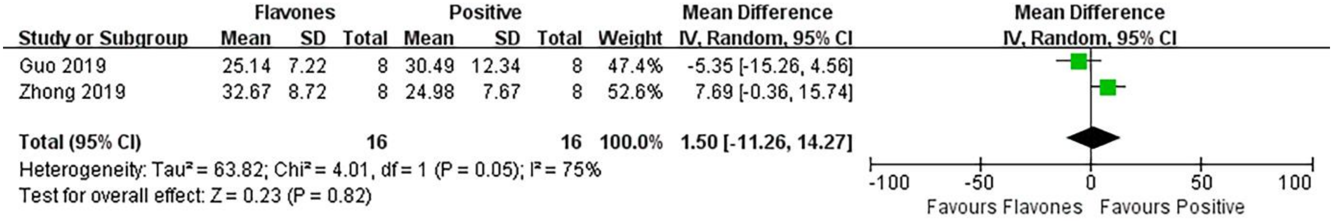

Figure S18. Forest for the IL-1β levels in the hippocampus (pg/ml) (compared with the positive group).

	Flavones			Positive				Mean Difference
Study or Subgroup	Mean	SD	Total	Mean	SD	Total	Weight	IV, Random, 95% CI
Li 2015a	66.47	22.86	6	80.39	18.84	6	39.4%	-13.92 [-37.62, 9.78]
Liu 2019a	39.52	9.04	6	30.24	10.49	6	60.6%	9.28 [-1.80, 20.36]
**Total (95% CI)**			**12**			**12**	**100.0%**	**0.14 [-22.07, 22.36]**

Heterogeneity: Tau² = 180.01; Chi² = 3.02, df = 1 (P = 0.08); I² = 67%
Test for overall effect: Z = 0.01 (P = 0.99)

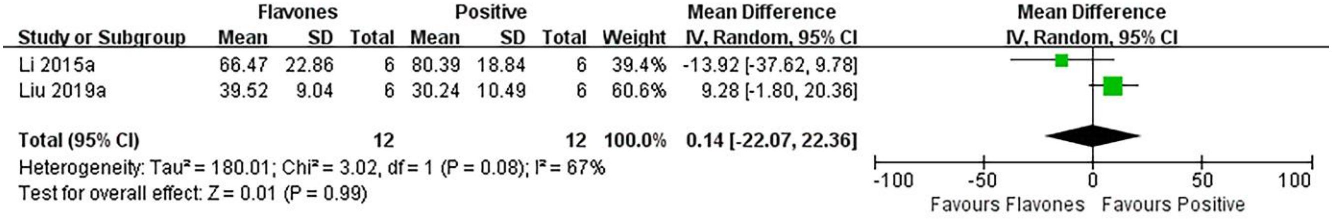

Figure S19. Forest for the IL-6 in the hippocampus level (compared with the positive group).

	Flavones			Positive				Mean Difference
Study or Subgroup	Mean	SD	Total	Mean	SD	Total	Weight	IV, Fixed, 95% CI
Guo 2019	277.95	25.64	8	291.54	38.49	8	16.7%	-13.59 [-45.64, 18.46]
Zhong 2019	34.1	16.16	8	30.67	12.93	8	83.3%	3.43 [-10.91, 17.77]
**Total (95% CI)**			**16**			**16**	**100.0%**	**0.59 [-12.50, 13.68]**

Heterogeneity: Chi² = 0.90, df = 1 (P = 0.34); I² = 0%
Test for overall effect: Z = 0.09 (P = 0.93)

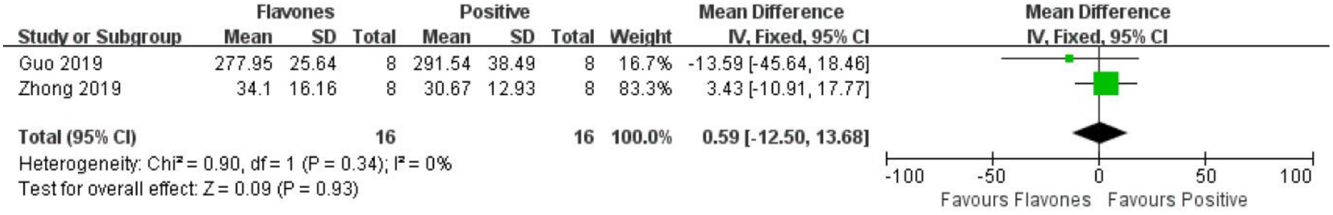

Figure S20. Forest for the TNF-α in the blood level.

	Flavones			Positive				Mean Difference
Study or Subgroup	Mean	SD	Total	Mean	SD	Total	Weight	IV, Fixed, 95% CI
Liu 2019a	61.54	7.33	6	57.69	6.3	6	63.1%	3.85 [-3.88, 11.58]
Zhong 2019	57.07	11.74	8	46.14	8.66	8	36.9%	10.93 [0.82, 21.04]
**Total (95% CI)**			**14**			**14**	**100.0%**	**6.46 [0.32, 12.61]**

Heterogeneity: Chi² = 1.19, df = 1 (P = 0.28); I² = 16%
Test for overall effect: Z = 2.06 (P = 0.04)

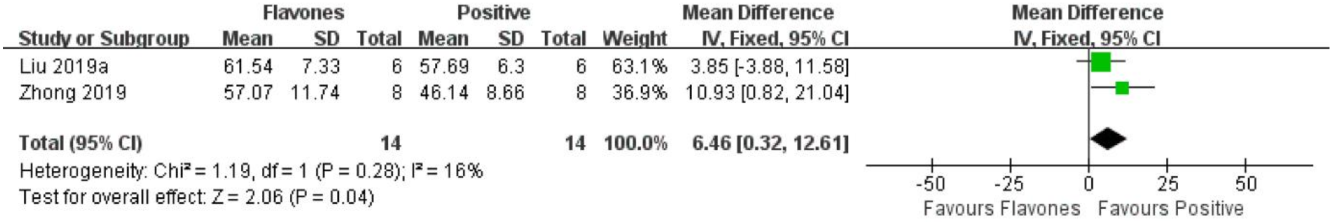

Figure S21. Forest for the TNF-α in the hippocampus level (pg/mg) (compared with the positive group).

	Flavones			Positive				Mean Difference
Study or Subgroup	Mean	SD	Total	Mean	SD	Total	Weight	IV, Random, 95% CI
Guo 2019	392.59	69.87	8	330.86	48.93	8	36.6%	61.73 [2.62, 120.84]
Zhong 2019	50.26	13.5	8	45.32	12.99	8	63.4%	4.94 [-8.04, 17.92]
**Total (95% CI)**			**16**			**16**	**100.0%**	**25.71 [-27.90, 79.32]**

Heterogeneity: Tau² = 1135.87; Chi² = 3.38, df = 1 (P = 0.07); I² = 70%
Test for overall effect: Z = 0.94 (P = 0.35)

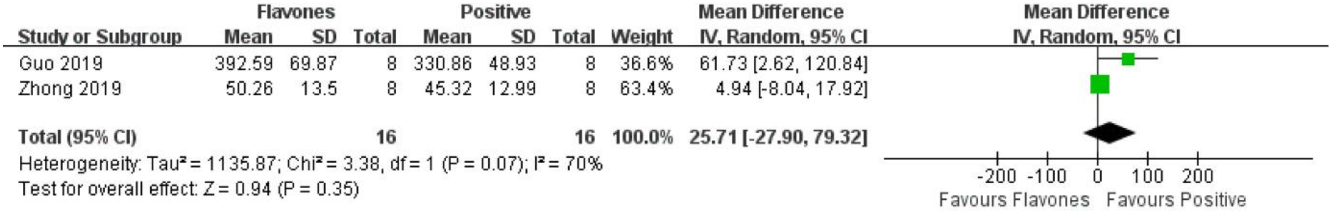

Figure S22. Forest for the TNF-α in the hippocampus level (pg/ml) (compared with the positive group).

	Flavones			Positive				Mean Difference
Study or Subgroup	Mean	SD	Total	Mean	SD	Total	Weight	IV, Random, 95% CI
Li 2015a	56.2	6.39	6	67.56	9.55	6	51.3%	-11.36 [-20.55, -2.17]
Liu 2019a	54.98	8.5	6	50.98	9.8	6	48.7%	4.00 [-6.38, 14.38]
**Total (95% CI)**			**12**			**12**	**100.0%**	**-3.88 [-18.92, 11.17]**

Heterogeneity: Tau² = 92.94; Chi² = 4.71, df = 1 (P = 0.03); I² = 79%
Test for overall effect: Z = 0.50 (P = 0.61)

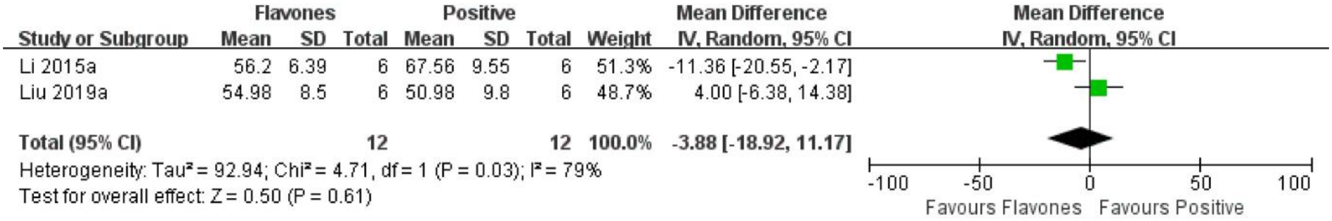

Figure S23. Forest for the NF-κB in the blood level.

	Flavones			Positive				Mean Difference
Study or Subgroup	Mean	SD	Total	Mean	SD	Total	Weight	IV, Random, 95% CI
Li 2015a	0.38	0.05	3	0.37	0.02	3	54.5%	0.01 [-0.05, 0.07]
Liu 2019a	0.53	0.1	6	0.39	0.07	6	45.5%	0.14 [0.04, 0.24]
**Total (95% CI)**			**9**			**9**	**100.0%**	**0.07 [-0.06, 0.20]**

Heterogeneity: Tau² = 0.01; Chi² = 4.90, df = 1 (P = 0.03); I² = 80%
Test for overall effect: Z = 1.07 (P = 0.29)

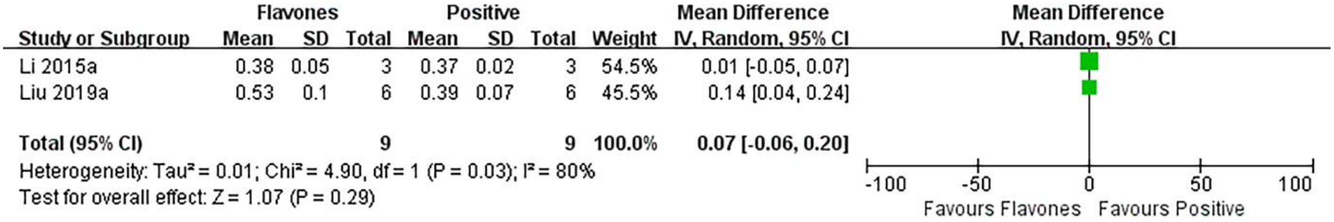

Figure S24. Forest for the BDNF levels (compared with the positive group).

Study or Subgroup	Flavones Mean	Flavones SD	Flavones Total	Positive Mean	Positive SD	Positive Total	Weight	Mean Difference IV, Fixed, 95% CI
Li 2015b	0.97	0.19	5	1.02	0.13	5	43.7%	-0.05 [-0.25, 0.15]
Lu 2019	1.46	0.4	8	1.12	0.42	8	11.0%	0.34 [-0.06, 0.74]
Weng 2016	1.17	0.23	8	1.09	0.17	8	45.3%	0.08 [-0.12, 0.28]
**Total (95% CI)**			**21**			**21**	**100.0%**	**0.05 [-0.08, 0.19]**

Heterogeneity: Chi² = 3.03, df = 2 (P = 0.22); I² = 34%
Test for overall effect: Z = 0.76 (P = 0.45)

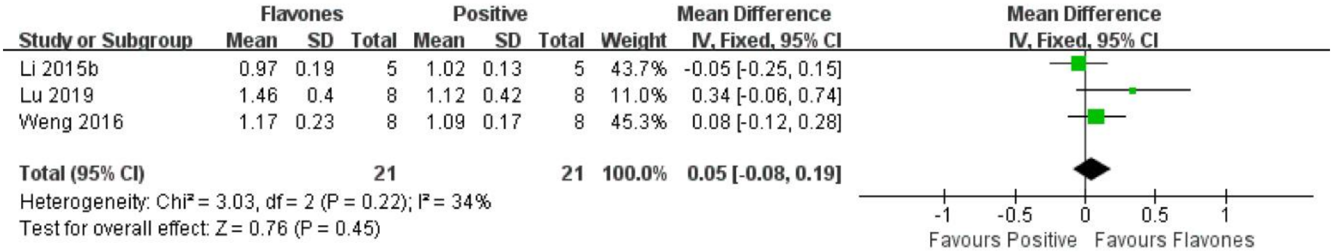

Figure S25. Forest for the IL-6 in the blood level (compared with the positive group).

Study or Subgroup	Flavones Mean	Flavones SD	Flavones Total	Positive Mean	Positive SD	Positive Total	Weight	Mean Difference IV, Fixed, 95% CI
Liu 2019a	54.59	7.08	6	42.52	6.25	6	68.6%	12.07 [4.51, 19.63]
Zhong 2019	50.47	13.22	8	45.56	9.25	8	31.4%	4.91 [-6.27, 16.09]
**Total (95% CI)**			**14**			**14**	**100.0%**	**9.82 [3.56, 16.09]**

Heterogeneity: Chi² = 1.08, df = 1 (P = 0.30); I² = 8%
Test for overall effect: Z = 3.08 (P = 0.002)

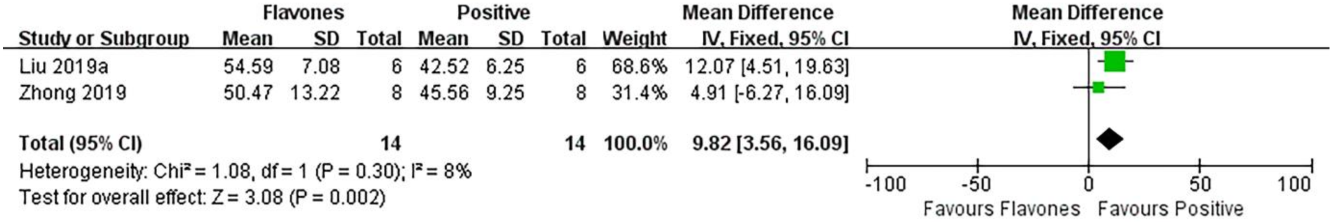

Figure S26. Forest plot for the effect of flavones on the sucrose preference test (compared with the positive group).

Study or Subgroup	Flavones Mean	Flavones SD	Flavones Total	Positive Mean	Positive SD	Positive Total	Weight	Mean Difference IV, Fixed, 95% CI
Alghamdi 2022	45.05	5.49	8	47.13	4.36	8	11.3%	-2.08 [-6.94, 2.78]
Guo 2019	76.33	12.25	12	71.65	16.19	12	2.0%	4.68 [-6.81, 16.17]
Jia 2021	81.35	4.58	10	75.56	3.89	10	19.2%	5.79 [2.07, 9.51]
Jin 2023	83.25	5.01	9	82.3	4.71	9	13.2%	0.95 [-3.54, 5.44]
Li 2015a	74.25	6.07	10	73.29	8.72	10	6.1%	0.96 [-5.63, 7.55]
Li 2015b	69	18.77	10	75.42	23.64	10	0.8%	-6.42 [-25.13, 12.29]
Liu 2019a	72.09	8.96	12	75.97	9.38	12	4.9%	-3.88 [-11.22, 3.46]
Liu 2019b	77	7.56	10	72.17	6.49	10	7.0%	4.83 [-1.35, 11.01]
Lu 2019	81.94	7.87	8	77.08	7.22	8	4.9%	4.86 [-2.54, 12.26]
Lu 2022	78.7	4.65	9	73.34	4.65	9	14.4%	5.36 [1.06, 9.66]
Ma 2023	81.45	12.89	10	79.87	10.93	10	2.4%	1.58 [-8.89, 12.05]
Sheng 2016	72.41	28.63	10	76.15	26.32	10	0.5%	-3.74 [-27.84, 20.36]
Weng 2016	75.09	18.82	8	79.68	15.71	8	0.9%	-4.59 [-21.58, 12.40]
Xiao 2021	81.89	20.28	9	83.04	22.17	9	0.7%	-1.15 [-20.78, 18.48]
Zhang 2019b	83.22	14.31	10	76.3	19.78	10	1.2%	6.92 [-8.21, 22.05]
Zhang 2019c	66.35	18.62	8	54.46	11.35	8	1.2%	11.89 [-3.22, 27.00]
Zhao 2020	80.55	7.27	10	75.45	8.85	10	5.3%	5.10 [-2.00, 12.20]
Zhe 2022	81.45	11.55	8	79.87	9.79	8	2.4%	1.58 [-8.91, 12.07]
Zhong 2019	61.89	12.59	8	79.42	13.36	8	1.6%	-17.53 [-30.25, -4.81]
**Total (95% CI)**			**179**			**179**	**100.0%**	**2.47 [0.84, 4.10]**

Heterogeneity: Chi² = 26.60, df = 18 (P = 0.09); I² = 32%
Test for overall effect: Z = 2.97 (P = 0.003)

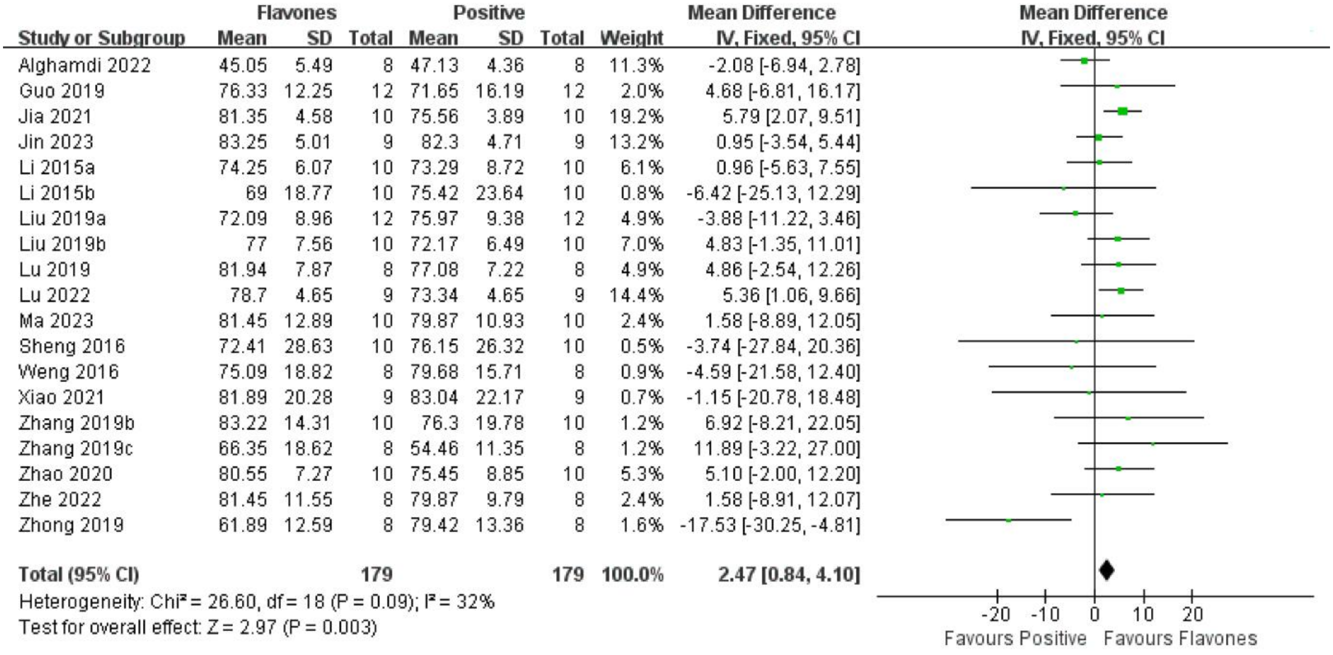

Figure S27. Forest for the CORT levels (compared with the positive group).

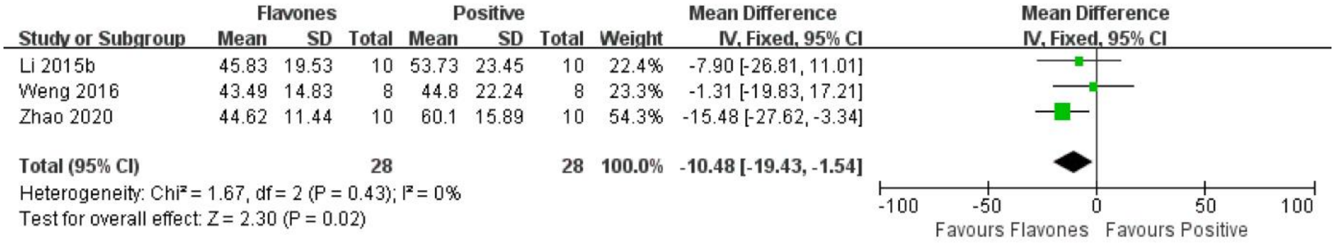

Figure S28. Subgroup analysis of pooled estimates of behavioral tests on the(a)tail suspension test (b)forced swimming test and(c)sucrose preference test.

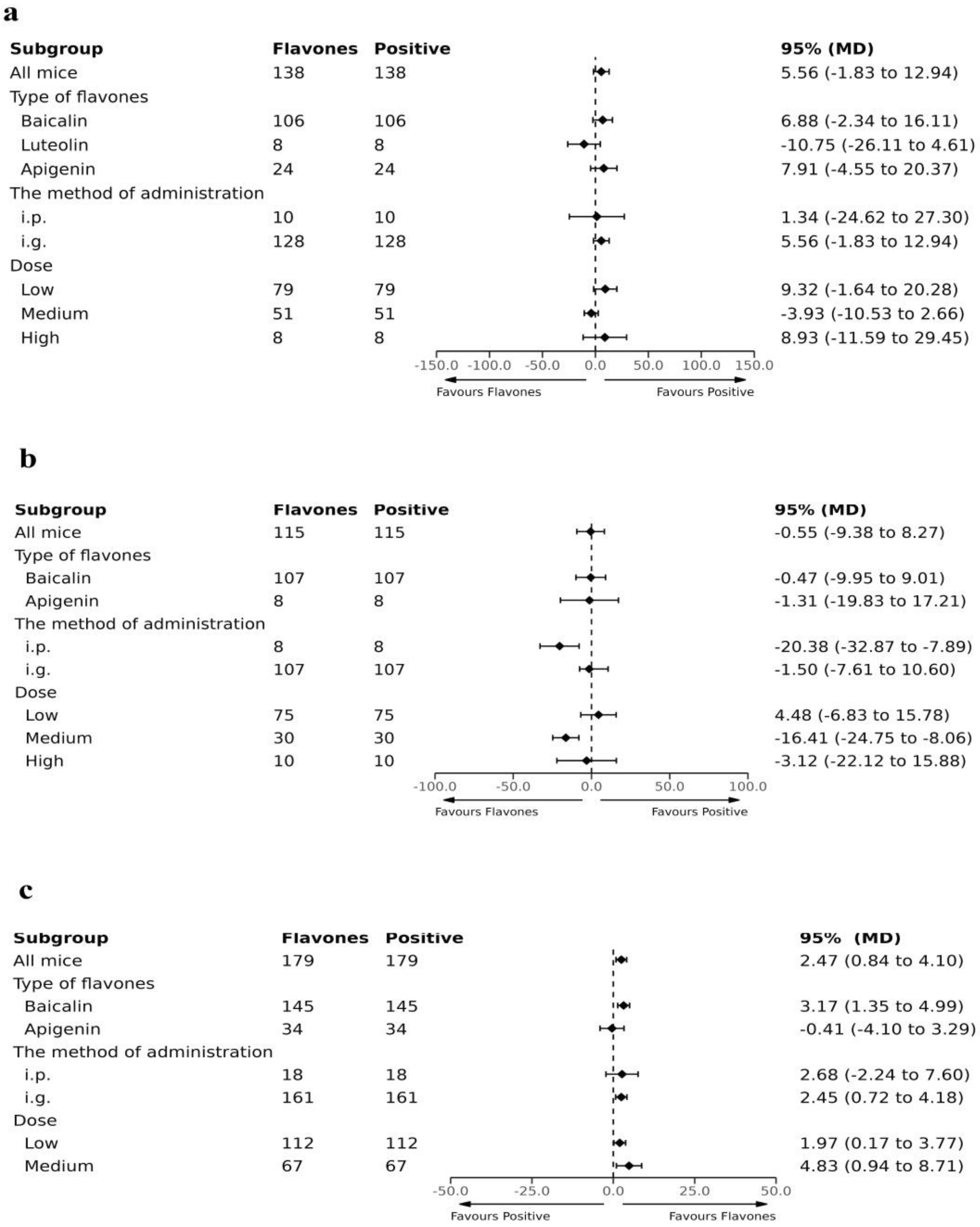

Figure S29. Sensitivity analyses of the tail suspension test.

	Flavones			Model				Mean Difference
Study or Subgroup	Mean	SD	Total	Mean	SD	Total	Weight	IV, Random, 95% CI
Alghamdi 2022	135.25	21.73	8	125.51	20.29	8	6.8%	9.74 [-10.86, 30.34]
Al-Yamani 2022	87.44	14.2	6	139.33	20.09	6	6.9%	-51.89 [-71.58, -32.20]
Can 2013	30.47	13.3	8	110.44	27.82	8	6.6%	-79.97 [-101.34, -58.60]
Jia 2021	78.06	42.94	10	128.4	17.06	10	5.3%	-50.34 [-78.98, -21.70]
Jin 2023	130.64	16.14	9	165.72	13.98	9	8.0%	-35.08 [-49.03, -21.13]
Li 2015a	66.11	27.43	10	147.59	34.54	10	5.6%	-81.48 [-108.82, -54.14]
Liu 2013	59	45.47	10	115.66	69.55	10	2.7%	-56.66 [-108.16, -5.16]
Liu 2019a	68.32	11.8	12	106	15.67	12	8.5%	-37.68 [-48.78, -26.58]
Liu 2022	107.38	36.97	6	197.63	19.36	6	4.6%	-90.25 [-123.64, -56.86]
Ma 2023	32.89	21.55	10	81.48	13.68	10	7.7%	-48.59 [-64.41, -32.77]
Xiao 2021	125.65	14.88	9	152.44	21.96	9	7.4%	-26.79 [-44.12, -9.46]
Zhang 2019b	40.88	21.61	10	93.99	33.15	10	6.0%	-53.11 [-77.64, -28.58]
Zhao 2020	66.67	10.21	10	99.36	9.64	10	8.9%	-32.69 [-41.39, -23.99]
Zhe 2022	89.35	14.91	8	131.74	13.27	8	8.0%	-42.39 [-56.22, -28.56]
Zhong 2019	77.45	22.39	8	107.79	17.66	8	6.9%	-30.34 [-50.10, -10.58]
**Total (95% CI)**			**134**			**134**	**100.0%**	**-44.50 [-54.52, -34.48]**

Heterogeneity: Tau² = 275.58; Chi² = 64.50, df = 14 (P < 0.00001); I² = 78%
Test for overall effect: Z = 8.70 (P < 0.00001)

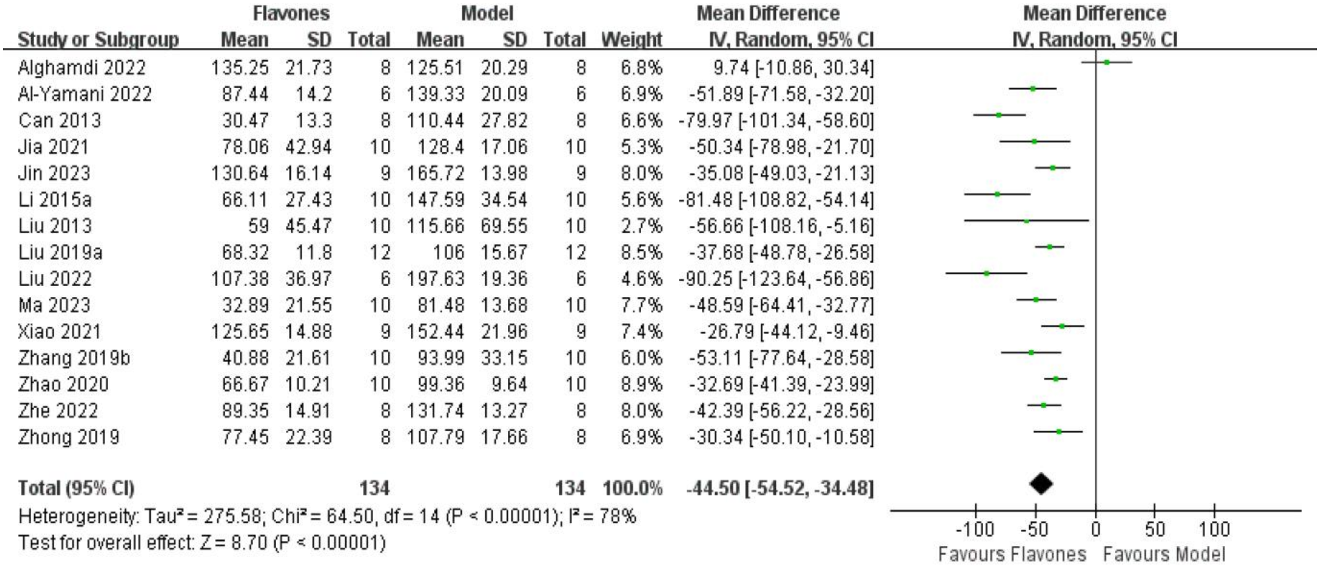

Figure S30. Sensitivity analyses of the forced swimming test.

	Flavones			Model				Mean Difference
Study or Subgroup	Mean	SD	Total	Mean	SD	Total	Weight	IV, Fixed, 95% CI
Li 2015b	45.83	19.53	10	89.91	32.07	10	3.0%	-44.08 [-67.35, -20.81]
Li 2022	88.62	15.32	8	112.4	20.28	8	5.2%	-23.78 [-41.39, -6.17]
Liu 2013	59.41	45.03	10	115.54	68.54	10	0.6%	-56.13 [-106.96, -5.30]
Liu 2019a	94.58	15.26	12	129.36	24.53	12	6.0%	-34.78 [-51.13, -18.43]
Liu 2019b	77	8.24	10	122.83	7.44	10	33.9%	-45.83 [-52.71, -38.95]
Liu 2022	70.7	25.11	6	108.37	19.43	6	2.5%	-37.67 [-63.07, -12.27]
Lu 2019	69.58	13.7	8	94.31	14.77	8	8.2%	-24.73 [-38.69, -10.77]
Lu 2022	117.39	15	9	151.23	28.08	9	3.7%	-33.84 [-54.64, -13.04]
Sheng 2016	42.43	23.67	10	84.17	22.63	10	3.9%	-41.74 [-62.04, -21.44]
Weng 2016	43.49	14.83	8	95.42	23.52	8	4.3%	-51.93 [-71.20, -32.66]
Zhao 2020	44.62	11.44	10	95.41	12.96	10	14.0%	-50.79 [-61.50, -40.08]
Zhe 2022	75.13	15.17	8	112.25	13.36	8	8.2%	-37.12 [-51.13, -23.11]
Zhong 2019	86.96	11.52	8	129.17	19.27	8	6.6%	-42.21 [-57.77, -26.65]
**Total (95% CI)**			**117**			**117**	**100.0%**	**-41.50 [-45.51, -37.50]**

Heterogeneity: Chi² = 16.97, df = 12 (P = 0.15); I² = 29%
Test for overall effect: Z = 20.32 (P < 0.00001)

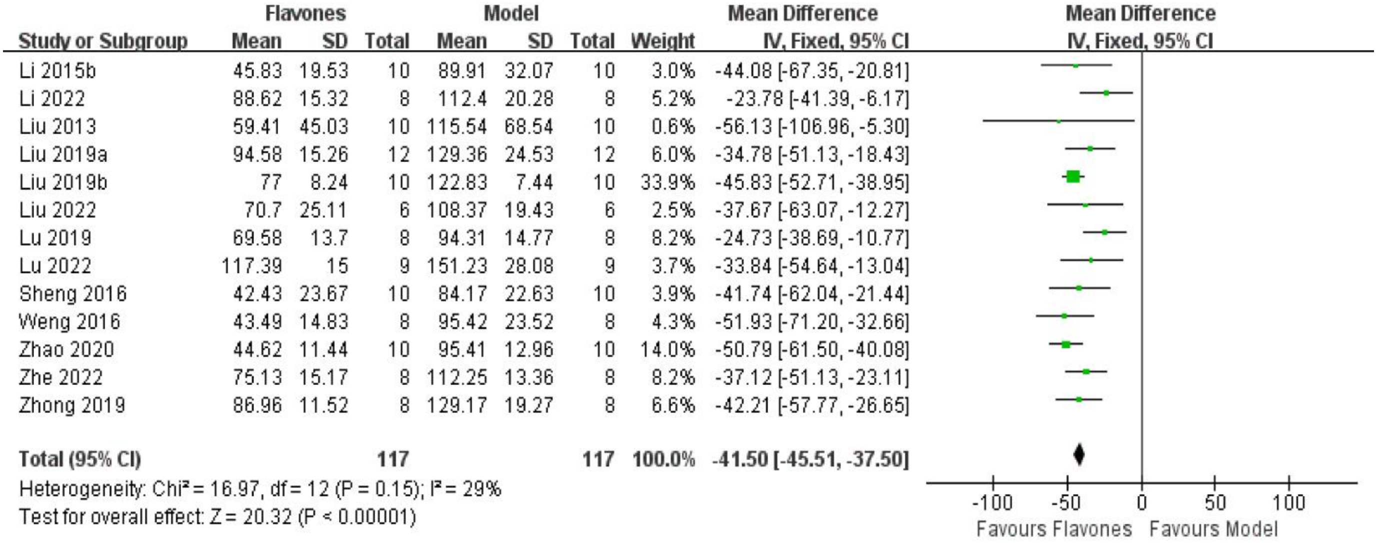

Figure S31. Sensitivity analyses of the sucrose preference test.

Study or Subgroup	Flavones Mean	Flavones SD	Flavones Total	Model Mean	Model SD	Model Total	Weight	Mean Difference IV, Random, 95% CI
Alghamdi 2022	45.05	5.49	8	14.02	3.57	8	6.9%	31.03 [26.49, 35.57]
Jia 2021	81.35	4.58	10	65.61	6.7	10	6.8%	15.74 [10.71, 20.77]
Jin 2023	83.25	5.01	9	69.39	11.01	9	6.1%	13.86 [5.96, 21.76]
Li 2015a	74.25	6.07	10	57.36	8.03	10	6.5%	16.89 [10.65, 23.13]
Li 2015b	69	18.77	10	58.72	21.3	10	3.6%	10.28 [-7.32, 27.88]
Li 2022	75.36	15.88	8	67.69	11.26	8	4.6%	7.67 [-5.82, 21.16]
Liu 2019a	72.09	8.96	12	75.97	9.38	12	6.2%	-3.88 [-11.22, 3.46]
Liu 2019b	77	7.56	10	55.17	10.66	10	6.0%	21.83 [13.73, 29.93]
Lu 2019	81.94	7.87	8	64.57	7.78	8	6.1%	17.37 [9.70, 25.04]
Lu 2022	78.7	4.65	9	65.94	5.13	9	6.9%	12.76 [8.24, 17.28]
Ma 2023	81.45	12.89	10	46.01	9.76	10	5.5%	35.44 [25.42, 45.46]
Sheng 2016	72.41	28.63	10	59.68	28.03	10	2.4%	12.73 [-12.10, 37.56]
Weng 2016	75.09	18.82	8	51.33	15.68	8	3.7%	23.76 [6.79, 40.73]
Xiao 2021	81.89	20.28	9	61.91	24.45	9	3.0%	19.98 [-0.77, 40.73]
Zhang 2019b	83.22	14.31	10	49.53	7.68	10	5.5%	33.69 [23.62, 43.76]
Zhang 2019c	66.35	18.62	8	42.77	15.65	8	3.8%	23.58 [6.73, 40.43]
Zhao 2020	80.55	7.27	10	60.44	8.25	10	6.3%	20.11 [13.29, 26.93]
Zhe 2022	81.45	11.55	8	71.57	10.7	8	5.3%	9.88 [-1.03, 20.79]
Zhong 2019	61.89	12.59	8	45.54	12.62	8	4.9%	16.35 [4.00, 28.70]
**Total (95% CI)**			**175**			**175**	**100.0%**	**17.94 [13.24, 22.65]**

Heterogeneity: Tau² = 78.53; Chi² = 101.31, df = 18 (P < 0.00001); I² = 82%
Test for overall effect: Z = 7.48 (P < 0.00001)

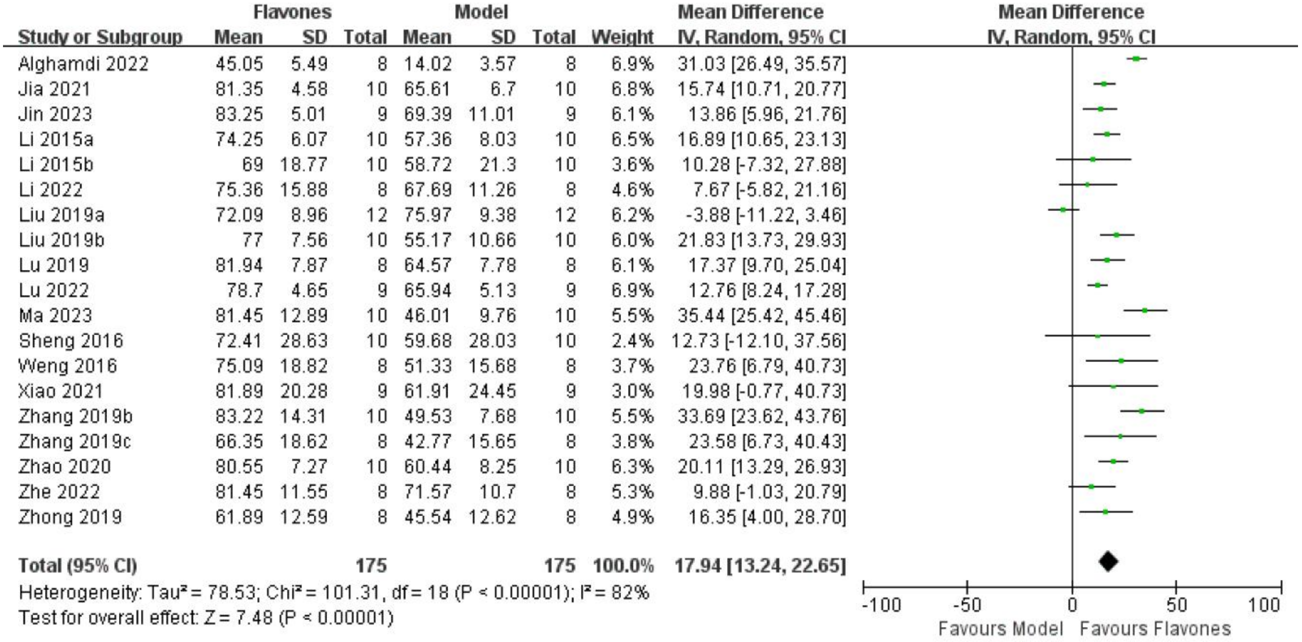

Figure S32. Sensitivity analyses of the tail suspension test (compared with the positive group).

Study or Subgroup	Flavones Mean	Flavones SD	Flavones Total	Positive Mean	Positive SD	Positive Total	Weight	Mean Difference IV, Random, 95% CI
Alghamdi 2022	135.25	21.73	8	125.51	20.29	8	6.7%	9.74 [-10.86, 30.34]
Al-Yamani 2022	87.44	14.26	6	77.44	19.94	6	7.0%	10.00 [-9.62, 29.62]
Can 2013	30.47	13.3	8	41.22	17.74	8	8.3%	-10.75 [-26.11, 4.61]
Jia 2021	78.06	42.94	10	71.17	29.64	10	4.1%	6.89 [-25.45, 39.23]
Jin 2023	130.64	16.14	9	84.89	18.63	9	8.1%	45.75 [29.65, 61.85]
Li 2015a	66.11	27.43	10	64.77	31.66	10	5.3%	1.34 [-24.62, 27.30]
Liu 2019a	68.32	11.8	12	64.92	14.5	12	9.9%	3.40 [-7.18, 13.98]
Ma 2023	32.89	21.55	10	31.68	20.29	10	7.4%	1.21 [-17.14, 19.56]
Xiao 2021	125.65	14.88	9	125.9	14.88	9	8.9%	-0.25 [-14.00, 13.50]
Zhang 2019b	40.88	21.61	10	39.17	18.36	10	7.6%	1.71 [-15.87, 19.29]
Zhao 2020	66.67	10.21	10	75.82	11.5	10	10.3%	-9.15 [-18.68, 0.38]
Zhe 2022	89.35	14.91	8	84.09	14.4	8	8.7%	5.26 [-9.10, 19.62]
Zhong 2019	77.45	22.39	8	66.71	11.46	8	7.7%	10.74 [-6.69, 28.17]
**Total (95% CI)**			**118**			**118**	**100.0%**	**5.39 [-2.65, 13.43]**

Heterogeneity: Tau² = 140.39; Chi² = 38.46, df = 12 (P = 0.0001); I² = 69%
Test for overall effect: Z = 1.31 (P = 0.19)

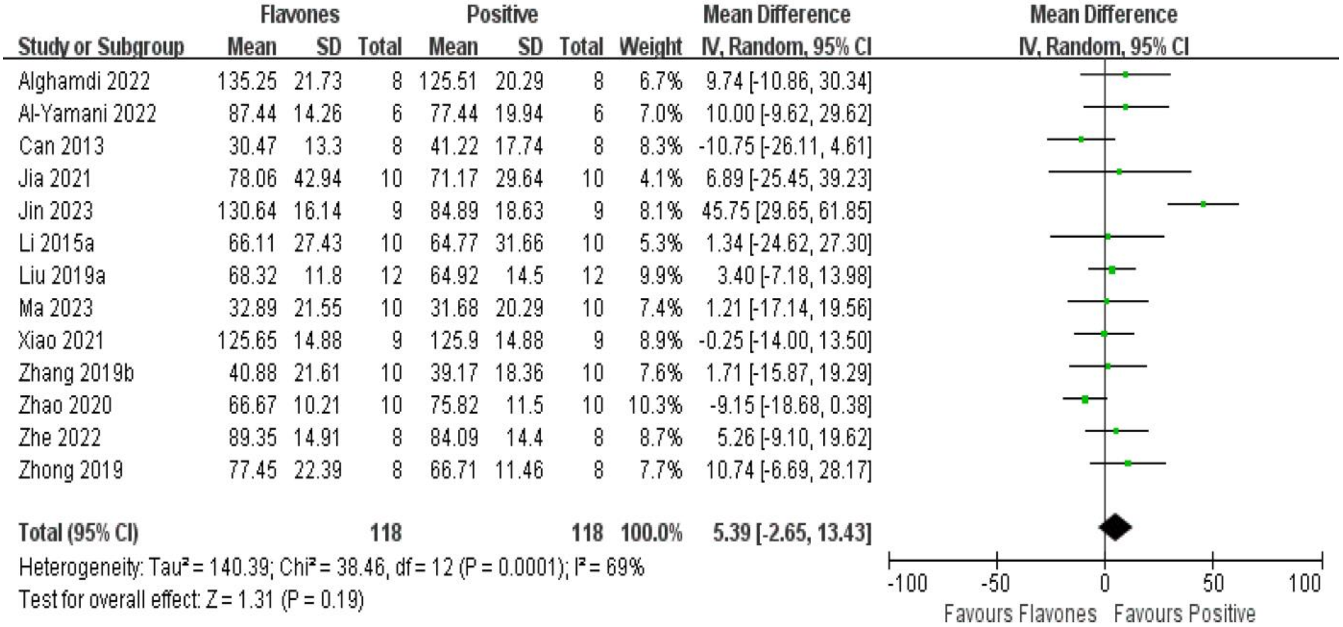

Figure S33. Sensitivity analyses of the forced swimming test (compared with the positive group).

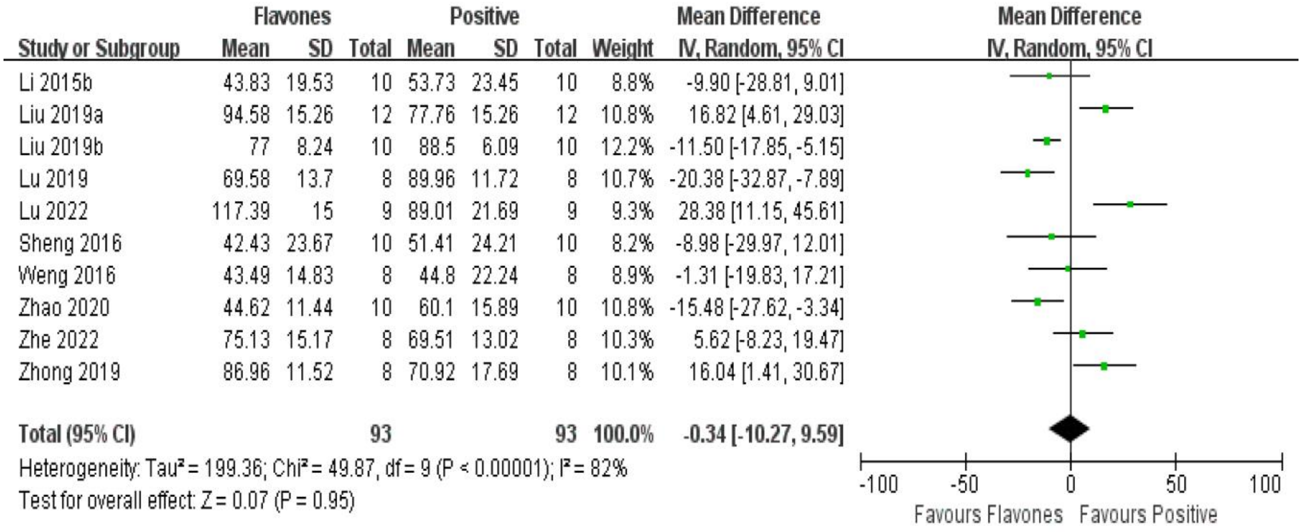

Figure S34. Sensitivity analyses of the sucrose preference test (compared with the positive group).

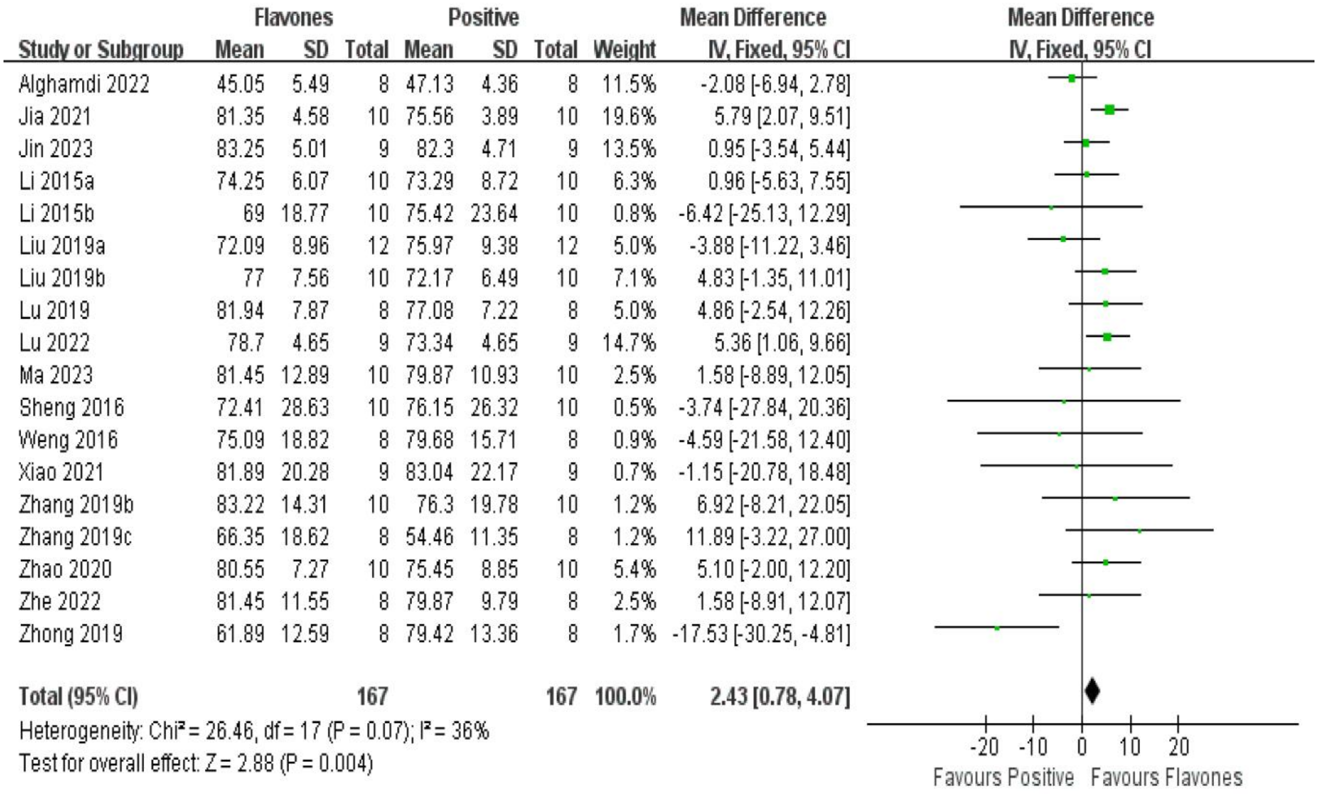

Figure S35. Trial sequential analysis for (a) tail suspension test (b) forced swimming test (c) sucrose preference test (d) OFT (crossing number) and (e) OFT (distance traveled) (compared with the positive group).

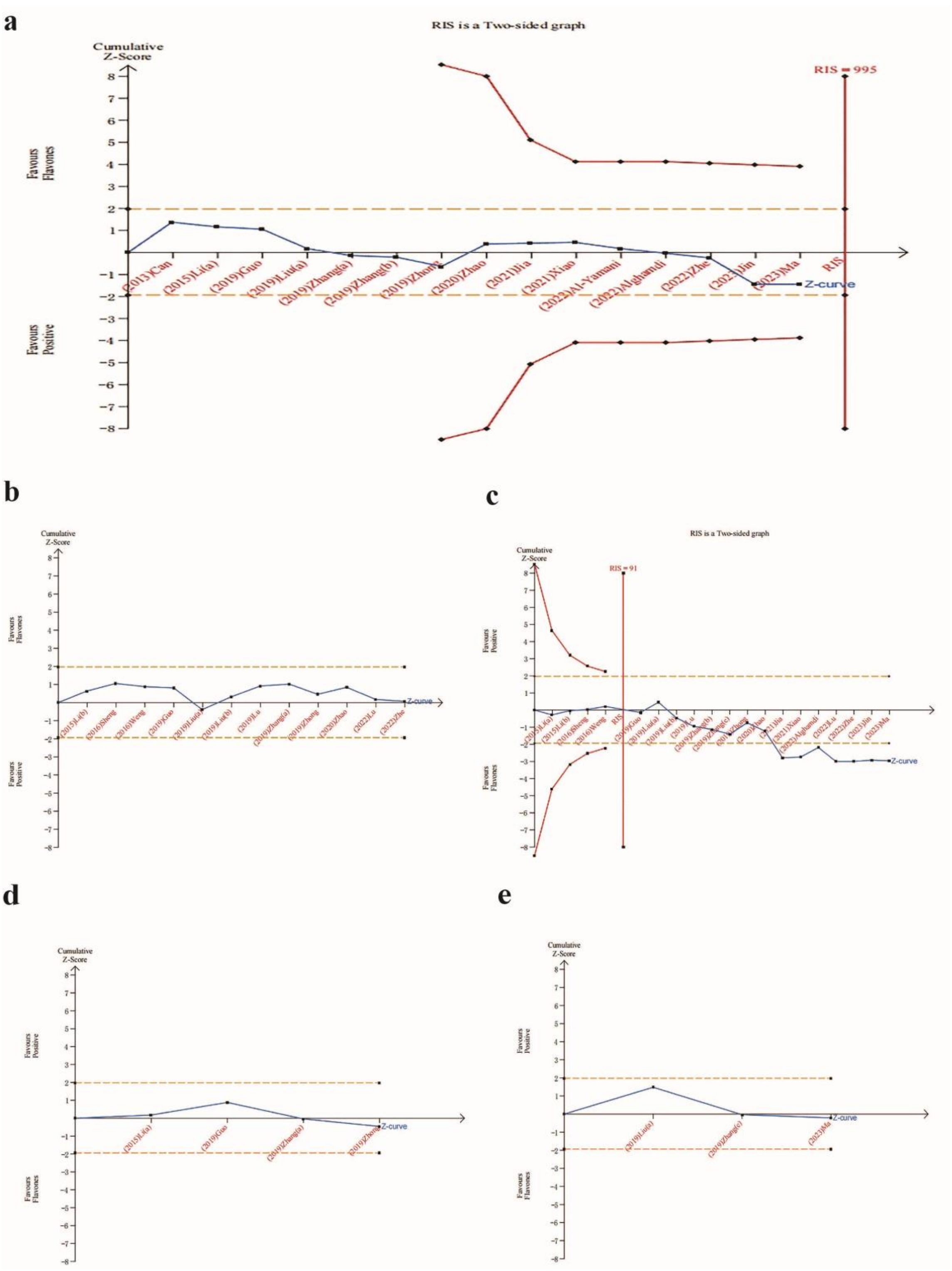

Supplement: Supplementary_Material.pdf [file IPHB_A_2467374_SM0179.pdf]
